# Quantifying the impact of surgical teams on each stage of the operating room process

**DOI:** 10.3389/fdgth.2024.1455477

**Published:** 2024-10-03

**Authors:** Adam Meyers, Mertcan Daysalilar, Arman Dagal, Michael Wang, Onur Kutlu, Mehmet Akcin

**Affiliations:** ^1^ Department of Industrial and Systems Engineering, University of Miami, Coral Gables, FL, United States; ^2^ Department of Anesthesiology, Perioperative Medicine, and Pain Management, Miller School of Medicine, University of Miami, Miami, FL, United States; ^3^ Department of Neurological Surgery, Miller School of Medicine, University of Miami, Miami, FL, United States; ^4^ DeWitt Daughtry Family Department of Surgery, Miller School of Medicine, University of Miami, Miami, FL, United States

**Keywords:** operating room, surgery, efficiency, case delay, duration, surgical team, human factors, linear mixed model

## Abstract

**Introduction:**

Operating room (OR) efficiency is a key factor in determining surgical healthcare costs. To enable targeted changes for improving OR efficiency, a comprehensive quantification of the underlying sources of variability contributing to OR efficiency is needed. Previous literature has focused on select stages of the OR process or on aggregate process times influencing efficiency. This study proposes to analyze the OR process in more fine-grained stages to better localize and quantify the impact of important factors.

**Methods:**

Data spanning from 2019-2023 were obtained from a surgery center at a large academic hospital. Linear mixed models were developed to quantify the sources of variability in the OR process. The primary factors analyzed in this study included the primary surgeon, responsible anesthesia provider, primary circulating nurse, and procedure type. The OR process was segmented into eight stages that quantify eight process times, e.g., procedure duration and procedure start time delay. Model selection was performed to identify the key factors in each stage and to quantify variability.

**Results:**

Procedure type accounted for the most variability in three process times and for 44.2% and 45.5% of variability, respectively, in procedure duration and OR time (defined as the total time the patient spent in the OR). Primary surgeon, however, accounted for the most variability in five of the eight process times and accounted for as much as 21.1% of variability. The primary circulating nurse was also found to be significant for all eight process times.

**Discussion:**

The key findings of this study include the following. (1) It is crucial to segment the OR process into smaller, more homogeneous stages to more accurately assess the underlying sources of variability. (2) Variability in the aggregate quantity of OR time appears to mostly reflect the variability in procedure duration, which is a subinterval of OR time. (3) Primary surgeon has a larger effect on OR efficiency than previously reported in the literature and is an important factor throughout the entire OR process. (4) Primary circulating nurse is significant for all stages of the OR process, albeit their effect is small.

## 1 Introduction

Improving operating room (OR) efficiency is a key factor in controlling or reducing surgical healthcare costs (1), which are significant. Aggregate surgical healthcare expenditures comprised 29% of aggregate healthcare expenditures in the United States in 2005, as computed by Muñoz et al. (2). Moreover, aggregate surgical expenditures were forecasted to grow from 4.6% of US GDP in 2005 to 7.3% of US GDP in 2025 (2). In a more recent study by Childers and Maggard-Gibbons (3), the mean cost of ambulatory OR time across California hospitals in fiscal year 2014 was $36.14 per minute with a standard deviation of $19.53 per minute. Cerfolio et al. (4) report a significantly higher cost of $150 per minute of OR time in the main campus ORs at New York University Langone Health. Even with financial considerations aside, improving OR efficiency will likely improve patient safety, experience, and outcomes, decrease patient wait time, increase OR throughput, and improve surgical team and staff satisfaction (5, 6).

Improving OR efficiency is a multifaceted problem, and several metrics have been investigated by researchers.^1^ A common approach to improving efficiency is to improve the utilization of the OR, that is, by minimizing both underutilization and overutilization (9, 10). Underutilization occurs when an OR lies unused due to cases being completed earlier than predicted, and overutilization occurs when an OR is used beyond its predicted or allotted time (5). Such inefficiencies are caused in large part by variability in OR time (11, 12), typically defined as the duration of time from when the patient is wheeled into the OR to the time the patient is wheeled out. Indeed, studies by Bokshan et al. (13) and Allen et al. (14) have shown OR time to be a significant driver of increased surgical costs. To reduce inefficiencies and associated costs, researchers have sought to identify the sources of variability in OR time. The primary conclusion in the literature is that procedure characteristics, namely, precise procedure type and type of anesthesia, are the main factors explaining the variation in OR time, followed by surgical team characteristics, primarily the surgeon (11, 12, 15, 16). Other factors such as patient characteristics (e.g., BMI) or other surgical team factors, such as the anesthesiologist, are generally found to be insignificant.

**Figure 1:**
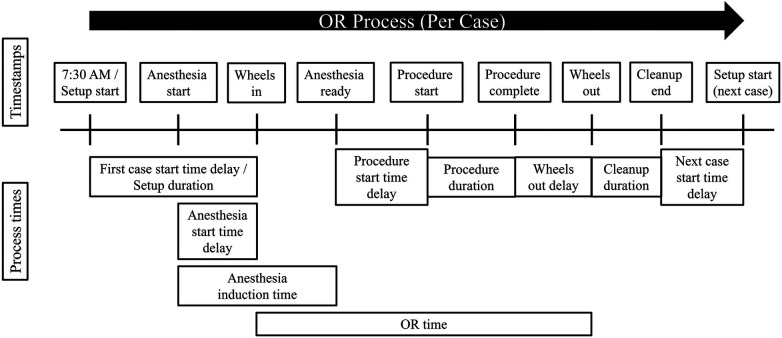
Visual depiction of the OR process, including timestamps and the span of time each OR process time covers. Formulas for each process time are given in Table 1.

OR time, however, is an aggregate quantity that encompasses several stages of the OR process, and it does not span the entire OR process (Figure 1). As such, it has the following potential downsides. First, OR time does not include all stages of the OR process. In this study’s dataset, which consists of timestamps taken from a surgery center located in a large academic hospital, OR time does not include room setup duration or room cleanup duration, nor any delays in starting the next case or beginning anesthesia induction. In addition, the dataset shows that anesthesia induction begins, on average, approximately two minutes before the patient is wheeled into the OR (i.e., two minutes before OR time begins). Thus, analyzing OR time alone will not allow for ascertaining the sources of variability in all stages of the OR process, and it may also contain some inaccuracies due to the starting and ending points of OR time not lining up with the activities in the OR process. Second, OR time itself covers several different stages of the OR process, including anesthesia induction, procedure duration, and delays in the procedure start time and in the time the patient is wheeled out after the procedure is completed. It is reasonable to hypothesize that the above four stages do not have the same sources of variability, or that shared sources of variability do not account for the same proportion of variability across all stages of the OR process. Therefore, this study’s approach is to segment the OR process into more fine-grained, homogeneous stages and assess the sources of variability within each stage.

Past studies have focused on other parts of the OR process besides OR time and the more fine-grained stages that comprise OR time, including surgical procedure duration, anesthesia-related times, start time delays, and turnover time (refer to Section 2.1). However, an effort to quantify the sources of variability across all fine-grained stages of the OR process is currently lacking. Based on timing markers obtained from a surgery center located in a large academic hospital, this paper quantifies the sources of variability in several OR process stages, including first case start time delay, setup duration, anesthesia induction time, procedure start time delay, procedure duration, wheels out delay, cleanup duration, and OR time (refer to Section 3.1). The focus of this paper is on quantifying the extent to which type of procedure and members of the surgical team - primary surgeon, responsible anesthesia provider, and primary circulating nurse - and their interactions explain the variation in the fine-grained stages of the OR process. By better understanding the influence of various important factors, stakeholders and researchers can better pinpoint where interventions to improve efficiency should be targeted.

The rest of this paper is organized as follows. Section 2 provides a literature review on previous approaches to assess or improve efficiency within different stages of the OR process. Section 3 describes the dataset, process times, statistical approach, and model selection. Section 4 describe the results of the statistical analysis, primarily providing a decomposition of variability for each process time. Section 5 discusses the primary findings of this study and comments on this study’s limitations and opportunities for future work. Section 6 provides concluding remarks. Additional tables and figures generated in this study are available in the Supplementary Material.

## 2 Research background

### 2.1 Related work in determining the factors driving the stages of the OR process

Numerous studies have investigated the various factors purported to cause or explain the variation in the OR process. Such work is motivated by the idea that, for OR efficiency to be improved, relevant stakeholders must first be informed about the primary factors driving OR inefficiencies. In addition, identifying the primary factors will allow for better predictive modeling, which in turn will allow for more accurate OR case scheduling to reduce OR underutilization and overutilization.

Variability in OR time (i.e., “wheels in” to “wheels out” time) is cited as a primary cause of inefficient OR utilization (11, 12). When a case lasts longer than planned, subsequent cases will either be delayed, potentially leading to OR overutilization, or cancelled, resulting in less OR revenue, patient dissatisfaction, and reduced quotas for surgical teams. When a case lasts shorter than expected, the OR will likely lie underutilized for some period of time, wasting resources. Exploring the factors that explain the variability in OR time, Dexter et al. (15) verified earlier findings, e.g., in Strum et al. (16), that reported the importance of three factors: precise procedure information, surgical team, and anesthetic type in predicting OR time. Eijkemans et al. (11) later identified additional factors, including the surgeon’s estimate of total surgical time, operation characteristics (e.g., number of separate procedures), and team characteristics (e.g., number of surgeons). van Eijk et al. (12) found that type of procedure is the overwhelming predictor of OR time variability, with surgeon having a small but significant effect and anesthesiologist having a negligible effect. Many studies show that patient characteristics (e.g., body mass index) have little effect (11, 12).

Some studies have investigated the sources of variability in other parts of the OR process and in more fine-grained stages. The most commonly examined stage is the (surgical) procedure duration, which is typically the longest stage that comprises OR time. For instance, Strum et al. (16) found the surgeon to be the most important source of variability in procedure duration, followed by anesthesia type. Patoir et al. (17) found surgeon characteristics, center location, and surgical procedure and patient characteristics accounted for much of the variation in procedure duration. Additional factors were explored in the literature, such as surgeon factors (e.g, team composition factors, such as the presence of residents) (18), factors that increase the expected duration (e.g., communication failures) by Gillespie et al. (19), and operational (e.g., OR assignment) and temporal (e.g., whether a case was started after 5:00PM) factors by Kayis et al. (9). However, many of the studies focusing on procedure duration, e.g., Strum et al. (16), Stepaniak et al. (18), and Kayis et al. (9), perform statistical analyses separately for each surgical speciality or coarse-grained category rather than considering holistically how the specific procedure type, as indicated by a fine-grained category such as the American Medical Association’s Current Procedure Terminology codes (refer to Section 3.2), accounts for the variation in procedure duration.

Other parts of the OR process explored in the literature are anesthesia-related times. For instance, Kougias et al. (20) found in their multivariate regression analysis that procedure type, anesthesia type, and BMI were statistically significant predictors of anesthesia induction time, while procedure type, anesthesia type, and operative case length were statistically significant predictors of anesthesia recovery time. van Veen-Berkx et al. (21) found that scheduling accuracy improved when looking at anesthesia-controlled time (ACT) as a proportion of total procedure time.^2^ Few studies, however, have examined the impact of various human factors involved in the OR process on anesthesia-related times, including anesthesiologists.

Other fine-grained stages of the OR process that have been explored include start time delays, such as procedure start time delay, (any) case start time delay, and first case start time delay. Does et al. (23) employed Six Sigma techniques (24) to identify poor planning and scheduling as the primary factor causing delays in the start times of surgical procedures. The authors noted that surgical specialty and anesthesia technique also influence start time delays. A review by Halim et al. (25) identified several factors that can improve start time, including financial incentives for staff, education strategies, perioperative protocols and systems, surgical team communication, the “golden patient” initiative,^3^ and the “productive operating theatre” scheme^4^ A more specific approach is to look only at delays in the first case of the day, with the justification being to mitigate the cascading effect a delay in the first case has on subsequent cases in the OR. Cox Bauer et al. (27) analyzed data across three high-volume urban hospitals and found that, for cases with a documented reason for delay, the physician was the most reported reason for delay at 52%, followed in descending order by anesthesia, patient, staff, other sources, and facility. The authors did perform a regression analysis finding patient age, occurrence of late arrival, department, and facility to be significant predictors of delay. However, neither approach gives a quantification of the overall impact of a predictor on first case start time delay. Other similar work has looked at more specific events such as delays in the start of a subsequent case when the preceding case was performed by a different surgeon (28) and remaining time to exit the OR after surgical closure begins (29).

An additional stage of the OR process explored in the literature is turnover time, which is the duration of time from when a patient is wheeled out until the next patient is wheeled in. Thus, turnover time is all the remaining time in the OR process not covered by OR time (Figure 1). Bhatt et al. (30) took a systems-level approach to improve turnover time, which focused on developing a consistent “room ready” designation to reduce variability, implementing parallel processing to ensure room readiness and patient readiness occur simultaneously, and improving perioperative communication. Cerfolio et al. (4) piloted a Performance Improvement Team, called “PIT Crew,” that performed lean processing and value mapping to improve efficiency in the turnover time period. Goldhaber et al. (31) reduced turnover times significantly by collecting more granular data within the turnover time period and displaying these data to teams for regular review and accountability. The turnover time period was further divided into the followings segments: wheels out time → cleanup start time → cleanup complete time → setup start time → time room is ready for patient → wheels in time. Few studies, however, have taken the approach of quantifying the factors that explain variation in turnover time or the stages that comprise the turnover time period.

### 2.2 State-of-the-practice methodologies for determining important factors

There are several approaches in the related literature that seek to identify the important factors accounting for the variation in the OR process. Primary methods found in the literature include performing basic statistical analysis, fitting known probability distributions to OR process times, utilizing regression approaches for inference or prediction, utilizing systems-level approaches for improving process efficiency, and, more recently, training machine learning models for prediction.

Traditional statistical analysis, such as descriptive statistics and hypothesis testing methods, is a fundamental approach to gaining insights from gathered datasets. Such analysis dates back many decades but is still utilized today, particularly with healthcare data, as it provides insights and an overview of process efficiency. Dexter et al. (22) used two-group, one-sided t-tests to determine if eliminating ACT would allow for additional cases to be completed during a typical 8-h workday. Martin and Langell (32) used Cuzick’s test for trend to evaluate whether pre-OR timeouts and performance pay improved on-time starts, OR utilization, and OR costs. Simmons et al. (33) was interested in determining if fine-grained CPT codes, compared to coarser-grained surgical specialties, would improve accuracy in surgical scheduling. They utilized the $I^{2}$ statistic and Levine’s test to assess heterogeneity in the means and variances, respectively, of ACTs and surgical-controlled times (SCTs).^5^ While traditional methods of statistical analysis can provide interpretable and meaningful summaries of data to answer questions of interest, such as determining whether differences in groups are significant following an intervention, further quantification capabilities are needed to assess the impact of factors on OR efficiency.

An early line of research involved finding distributions with a good fit to OR process time data. A main contributing paper in this approach is that of Strum et al. (34) in which the authors recommended using the lognormal distribution to model surgical procedure times. Stepaniak et al. (35) mostly corroborated the findings of Strum et al. (34), but Kayis et al. (9) found the lognormal distribution did not generally fit surgery duration well at the procedure level. Joustra et al. (36) more comprehensively fit a number of hazard models. However, as mentioned in Joustra et al. (36), such methods are less concerned with identifying the factors contributing to OR efficiency and more concerned with prediction.

Regression models, on the other hand, do allow for evaluating sources of variability in OR process times. Strum et al. (16) employed main-effects ANOVA modeling with the logarithm of surgical time and total procedure time as separate responses and found primary surgeon and type of anesthesia to be important predictors of variability. Does et al. (23) and Stepaniak et al. (18) also utilized ANOVA models to assess the importance of select factors on start time delays and surgical procedure times. Regression modeling is similarly used to identify factors that influence OR process times. Linear regression is especially utilized for this purpose, such as in Silber et al. (37), Ying Li and Huang (38), Gillespie et al. (19), and van Veen-Berkx et al. (21). Linear regression models also have added functionalities over ANOVA models, such as regularization techniques to avoid overfitting or to perform variable selection, e.g., LASSO used in Wang et al. (39), and incorporating nonlinear terms such as in Wang et al. (40).

The literature above utilizing linear regression methods tends to treat all factors as fixed effects. However, in a fixed effects setting, when certain units, e.g., surgeons, have few observations, parameter estimates may have high sample-to-sample variability. Thus, the parameter estimates may vary substantially from dataset to dataset, implying that the model built on a given dataset may not be reliable (41). In addition, fixed effects models require dummy variables to be created for each unit (e.g., each surgeon), and a coefficient must be estimated for each unit. If a factor contains many units (this study’s dataset contains over one hundred surgeons), then estimating a large number of coefficients reduces the model’s degrees of freedom, diminishes the model’s power, and increases the standard errors of the coefficient estimates (41). Furthermore, the present study is not concerned with estimating the effects of individual surgeons, anesthesiologists, etc., but rather the effect of these groups as a whole. For such reasons, previous papers in assessing the effects of different factors on OR process times have employed linear mixed model (LMM) approaches, which incorporate both fixed and random effects, with great success, e.g., Dexter and Ledolter (42), Eijkemans et al. (11), van Eijk et al. (12). This paper also takes an LMM approach for the above reasons.

More recently, machine learning (ML) has become a popular method for predicting quantities in the OR process. Master et al. (43) found that regression tree methods, such as gradient boosted regression trees, outperformed historical averaging, surgeon expert predictions, and other ML methods in the literature when predicting pediatric surgical durations. ML methods combined with surgeon predictions were also among the top-performing methods in Master et al. (43). Other research has used ML to improve predictions of OR process times (44–48). While ML methods may improve prediction, Wang and Dexter (49) notes that implementing ML software to increase prediction accuracy will not increase productivity unless accompanied by more allotted case time in a typical workday. More importantly, the objective of this paper is to quantify the impact that various human factors have on OR efficiency. LMMs allow for quantifying the proportion of variance explained in a response by each factor of interest. ML methods do have some options for determining similar values of impact, including variable importance in classification and regression trees (CART) (45) and Shapley additive explanations (SHAP) values (46). However, variable importance metrics may not correlate well with model variance explained by features (50), particularly when the model overfits the data on which it’s trained, which is a common issue with CART methods (43). The variable importance values are also typically reported in a relative fashion (to other variables) and thus do not provide an absolute assessment of the impact a factor has on a response. SHAP values may provide a better alternative in these regards, however they are not as well-established as linear regression-based metrics and may have issues as feature importance metrics (51).

## 3 Materials and methods

### 3.1 Dataset and subjects

Data spanning from January 2, 2019 to June 30, 2023 were obtained from a surgery center in the University of Miami Hospital. The surgery center incorporates six operating rooms and a dedicated preoperative area and postoperative recovery unit. The dataset originally contained 12,375 cases, before data cleaning was performed (detailed below). The dataset included the following timestamps: setup start time, anesthesia start time, wheels in time (i.e., when the patient enters the OR), anesthesia ready time, procedure start time, procedure complete time, wheels out time (i.e., when the patient exits the OR), and cleanup end time.^6^

This study examined various critical stages of the OR process rather than focusing solely on one stage or on aggregate process times encompassing several stages. The OR process times explored in this study included first case start time delay, setup duration, anesthesia induction time, procedure start time delay, procedure duration, wheels out delay, and cleanup duration.^7^ Each OR process time was defined as the elapsed time between two timestamps as described in Table 1. Figure 1 depicts the timestamps and process times.

**Table 1:** Formulas for calculating each OR process time and number of cases after individual data cleaning for each process time.

Process time	Formula	Nbr. of cases
First case start time delay	Wheels in – 7:30/8:30 AM*	3,543
Setup duration	Wheels in – setup start	4,681
Anesthesia induction time	Anesthesia ready – anesthesia start	5,480
Procedure start time delay	Procedure start – anesthesia ready	11,357
Procedure duration	Procedure complete – procedure start	11,501
Wheels out delay	Wheels out – procedure complete	11,326
Cleanup duration	Cleanup end – wheels out	4,800
OR time	Wheels out - wheels in	11,467

* 7:30 AM is the day’s start time for Monday, Tuesday, Wednesday, and Friday, and 8:30 AM is the start time for Thursday.

There is a strong focus in previous literature on the aggregate quantity, OR time, defined as the elapsed time between when the patient is wheeled into the OR to when the patient is wheeled out. It is hypothesized that the factors driving an aggregate quantity such as OR time, which covers various stages of the OR process (Figure 1), would not necessarily be identical across all stages comprising OR time, nor that shared sources of variability in OR time would explain the same proportion of variation in each stage comprising OR time. To evaluate these hypotheses, OR time was also included as a process time for comparison to the other seven process times.

This study’s statistical analysis (refer to Section 3.2) involved building separate regression models using each OR process time as a univariate response for a total of eight models. The subset of cases containing errors corresponding to one process time were not necessarily the same as the subset of cases containing errors for a different process time. Then, because separate models were developed for each process time, the choice was made to clean the data separately for each process time, maximizing the amount of data available for each model. Data cleaning involved removing any cases with missing data, outliers, or errors. In addition, any process time labeled as a “delay” only included delay times that were positive. For instance, if the first case started on or before the day’s start time, e.g., 7:30 AM, then this case was removed as there was no “delay” in the commencement of the first case. After removal of such cases for first case start time delay, the number of cases available for fitting the statistical model was 3,543 cases (Table 1). If instead the choice was made to remove the same subset of cases for all process times, then while each of the eight models would have a common pool of data, the data size would be significantly reduced and the results would not be as robust. The number of cases available after data cleaning for each process time is provided in Table 1.

**Figure 2:**
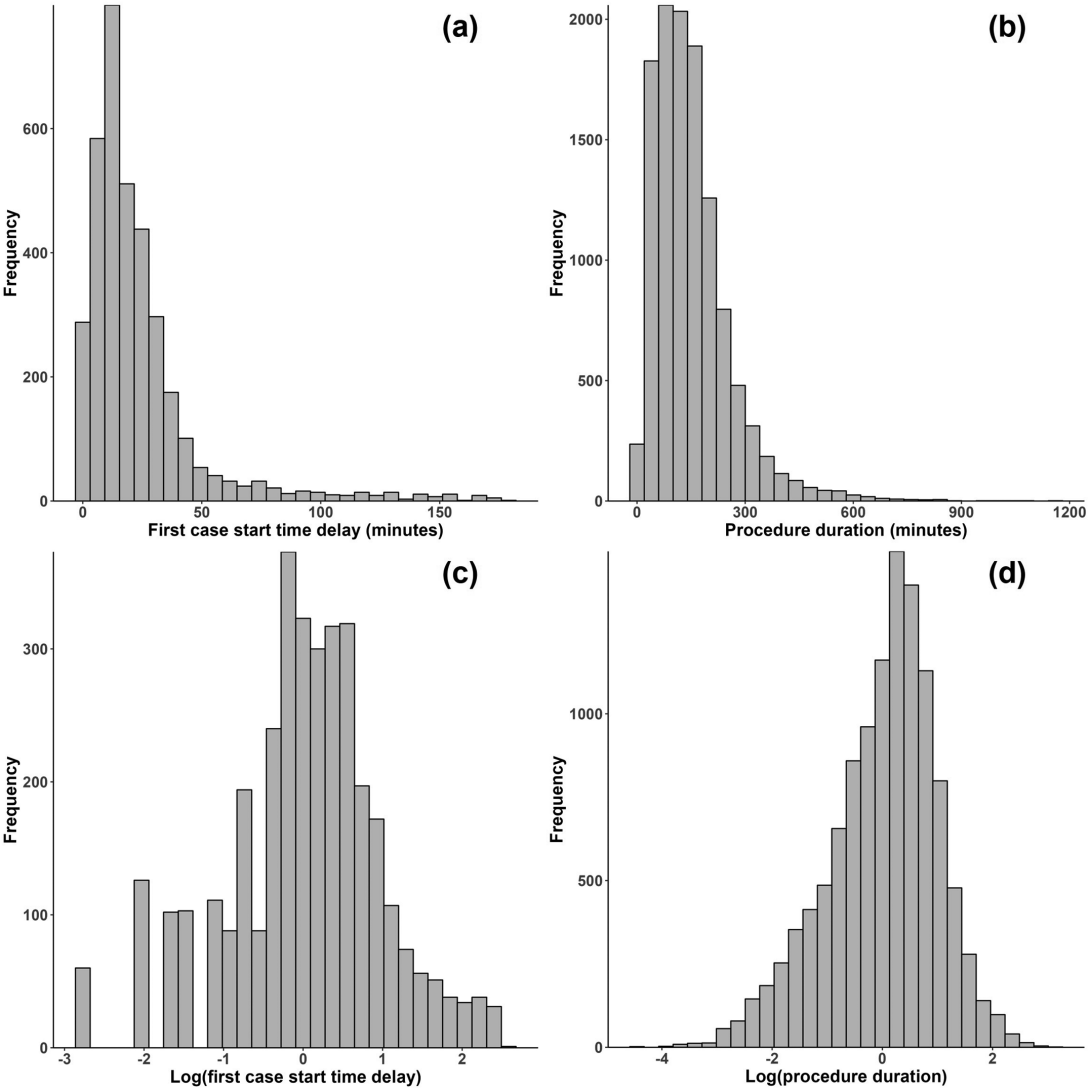
Histograms for **(a)** first cast start time delay and **(b)** procedure duration before transformation and **(c)** first case start time delay and **(d)** procedure duration after a log transformation.

All the OR process times exhibited right skewness. For instance, Figures 2(a,b) shows the original distributions of first case start time delay and procedure duration, where the right skewness is evident.^8^ To address this, a common approach in the relevant literature is to use a logarithm transformation. Eijkemans et al. (11) and van Eijk et al. (12) used the log transformation on OR time, and Strum et al. (34) and Stepaniak et al. (35) showed that OR time and procedure duration follow lognormal distributions, implying that log-transforming these process times will approximately yield a normal distribution more appropriate for linear regression modeling methods. Does et al. (23) were concerned with reducing start time delays of procedures, and to address right skewness they opted for a more thorough Box-Cox transformation. However, the optimal choice for the parameter λ in the Box-Cox tranformation was found to be zero in Does et al. (23), which is simply the log transformation. This study investigated several transformations, including log, square root, Box-Cox, and more. For many of the process times, the log transformation was not “optimal” in the sense of producing a distribution that most closely fits a normal distribution relative to all other transformations, however it was near-optimal for all process times. Moreover, given that the previous literature concluded the log transformation is appropriate for several OR process times and that the log transformation has better interpretability (in contrast to, e.g., the Box-Cox transformation), the logarithm was used to transform all process times in this study.

### 3.2 Statistical analyses

The primary objective of this study was to quantify the extent to which the variability observed in each OR process time could be attributed to four key factors: type of procedure, primary surgeon, responsible anesthesia provider, and primary circulating nurse. These factors will henceforth be referred to as “procedure,” “surgeon,” “anesthesiologist,” and “circulator,” respectively. Such analyses can provide a more precise account and quantification of the impact each factor has on each fine-grained stage of the OR process.

To quantify sources of variability in the OR process times, a linear mixed model (LMM) approach was used. An LMM was built separately for each of the eight process times, so that the sources of variability for each stage of the OR process could be assessed and quantified. The primary factors of interest, i.e., procedure, surgeon, anesthesiologist, and circulator were treated as random effects. Table 2 shows the number of levels of each factor that occurs in each process time’s corresponding dataset (after data cleaning).

**Table 2:** Number of levels of each random effect in the cleaned dataset for each OR process time.

OR process time	Random effect			
	Procedure	Surgeon	Anesthesiologist	Circulator
First case start time delay	439	106	78	112
Setup duration	568	106	76	114
Anesthesia induction time	633	118	77	117
Procedure start time delay	820	131	82	130
Procedure duration	827	132	82	131
Wheels out delay	818	131	82	130
Cleanup duration	519	107	79	121
OR time	823	132	82	131

The four primary factors were treated as random effects for multiple reasons. First, treating a factor as a random effect allows for estimating the factor’s variance and proportion of variance explained in the response (i.e., process time). Second, Table 2 shows that each of the four primary factors has many levels, and treating each as a fixed effect would require estimating tens to hundreds of coefficients, reducing the degrees of freedom in the model. This study is also not concerned with, e.g., a particular surgeon’s effect, but rather the impact of the group of surgeons as a whole. Third, the procedures, surgeons, anesthesiologists, and circulators included in the dataset do not necessarily encompass the entire populations of these factors. Thus, treating the factors as random effects allowed for accomplishing this study’s research objective and was an appropriate choice given the dataset. Note that only random intercepts were used in the LMMs.

Procedure was categorized based on the American Medical Association’s Current Procedure Terminology (CPT) codes (52). Several past studies have identified the importance of categorizing procedures with high granularity, e.g., with CPT codes, rather than with low granularity, e.g., with surgical specialities such as neurosurgery, gynecology, etc. (33, 34, 38). In particular, a recent study by Simmons et al. (33) examined over 30,000 surgical cases in an academic hospital and found that both the mean and variance of ACT and SCT varied significantly between CPT codes within specialities. Their results suggest that the use of more granular categories, specifically CPT codes, will enhance the accuracy of subsequent analysis and scheduling. Accordingly, this study used the primary CPT code for each case as the procedure type.

Other factors were available in the University of Miami Hospital’s database that could influence the process times. Domain expertise of this study’s authors was used to select the factors believed to impact OR process efficiency. Six factors were included; they are shown in Table 3. “Position,” for instance, was included as a proxy measure of the seniority and expertise of the primary surgeon. More experienced and senior surgeons were expected to be more efficient and consequently have a positive impact on OR efficiency. The six factors were treated as fixed effects for the following reasons. First, the factors were of less interest in this study and were expected to only marginally improve the model. The objective of this study was to quantify the sources of variability in the process times, focusing on procedure, surgeon, anesthesiologist, and circulator. Second, every factor had no more than five levels, with the exception of the number of procedures, which had thirty-two possible levels.^9^ Third, the levels of the factors were exhaustive of the population, whereas the levels of the random effects were only a subset of their respective populations.

**Table 3:** Description of fixed effects used in the LMMs.

Factor	Levels	Description
Number of procedures	0, 1,…, 31	Number of procedures performed in a surgical case.
Number of panels	1, 2,…, 5	Number of panels in a surgical case, where a “panel” is defined as a grouping of surgical procedures performed together.
Procedure level	None, I, II, III, IV	Indicates surgical complexity of case.
Cancer/noncancer	Cancer, noncancer	Indicates whether procedures were cancer-related or not.
Position	Assistant, associate, professor	Position of primary surgeon in academic hospital.
Patient class	Emergency, hospital ambulatory surgery, inpatient, surgery admit	Admission status of patient.

As stated previously, LMMs were separately built for each of the eight process times.^10^ Before model selection was performed, a univariate analysis of each random effect was conducted to quantify the improvement in each model by the addition of a single random effect. Two base models were used - one consisting of a fixed intercept and the other a fixed intercept plus all six fixed effects. To each base model, a single random effect was added and the adjusted intraclass correlation coefficient (ICC) was calculated for each random effect, given by

(1) $${\text{ICC}}_{\text{( adj) }}=\frac{\sigma_{\alpha }^{2}}{\sigma_{\alpha }^{2}+\sigma_{\epsilon }^{2}},$$

where $\sigma_{\alpha }^{2}$ refers to the variance of the random effect. ${\text{ICC}}_{\text{( adj) }}$ may be interpreted as the proportion of variance explained in the logarithm of the process time by the random effect, after controlling for the fixed effects.

After univariate analysis, multivariate analysis was performed to assess the impact of each random effect (in the presence of other significant random effects) on the process time and to control for fixed effects. Model selection proceeded as follows.^11^

First, a base model was developed, given by

(2) $$\begin{array}{cc} & y_{i}=\beta_{0}+X_{i}\beta +\alpha_{\;j[i]}+\epsilon_{i},\\ & \alpha_{\;j}∼N(0,\sigma_{\alpha }^{2}),\\ & \epsilon_{i}∼N(0,\sigma_{\epsilon }^{2}), \end{array}$$

where i=1,…,n and j=1,…,J are the indices of the observations and procedure levels, respectively, $y_{i}$ represents the ith observation of the logarithm of the respective process time, $\beta_{0}$ is the fixed intercept, β is the vector of fixed slopes, $X_{i}$ is the vector of the ith observations of all variables associated with the fixed effects (Table 3),^12^ $\alpha_{\;j[i]}$ is a random intercept for procedure, j[i] denotes to which procedure the ith observation belongs, $\epsilon_{i}$ is the error term, and $\sigma_{\alpha }^{2}$ and $\sigma_{\epsilon }^{2}$ are the variances of the random effect and error, respectively.

Second, note that the base model in Equation 2 only includes a random intercept for procedure. Procedure was previously found in multiple studies to be the primary source of variability in various OR process times (11, 12, 16, 35). Thus, with procedure ostensibly explaining much of the variation in the process times, it was reasonable to begin the base model with only procedure as a random intercept. Each additional random effect was subsequently and cumulatively added to determine if the additional random effect should be retained in the final model. A chi-squared test was used to determine the significance^13^ of a model with one additional random effect compared to the (previous) model without the random effect. Akaike information criterion (AIC) was also reported as it penalizes the addition of more terms to the LMM. However, the chi-squared test was solely used for determining which random effects to keep, since AIC is more appropriate for prediction which is not the objective of this study.

Third, fixed effects were individually examined to determine whether each should be retained in the final model for each process time. For a given process time, a new base model was formed by adding all significant random effects found in step two above to Equation 2. Each of the six fixed effects were individually removed from the new base model, while retaining all other fixed effects, and chi-squared tests were performed and AIC values were computed. If the new base model (containing all fixed effects and significant random effects from step two above) was found significant over the new base model without the individual fixed effect (according to the chi-squared test), then the fixed effect was retained for the final model.

Fourth, the final model for a given process time was formed by adding all significant random effects from step two above and removing all fixed effects according to the procedure described in step three above. To assess the impact of each random effect retained in the final model, ${\text{ICC}}_{\text{( adj) }}$ in Equation 1 was calculated for each random effect. In addition, model ICC values were calculated to give the overall proportion of variance explained in the logarithm of the process time by all random effects. Both unadjusted and adjusted model ICC values were reported. The unadjusted model ICC, denoted ${\text{ICC}}_{\text{LMM}}$, and adjusted model ICC, denoted ${\text{ICC}}_{\text{LMM( adj) }}$, are given by

(3) $${\text{ICC}}_{\text{LMM}}=\frac{\sigma_{\text{r}}^{2}}{\sigma_{\text{r}}^{2}+\sigma_{\text{\textbackslash{},f}}^{2}+\sigma_{\epsilon }^{2}}\;\text{and}\;{\text{ICC}}_{\text{LMM( adj) }}=\frac{\sigma_{\text{r}}^{2}}{\sigma_{\text{r}}^{2}+\sigma_{\epsilon }^{2}},$$

where $\sigma_{\text{r}}^{2}$ and $\sigma_{\text{\textbackslash{},f}}^{2}$ are the variances explained by all random and fixed effects, respectively.

## 4 Results

**Figure 3:**
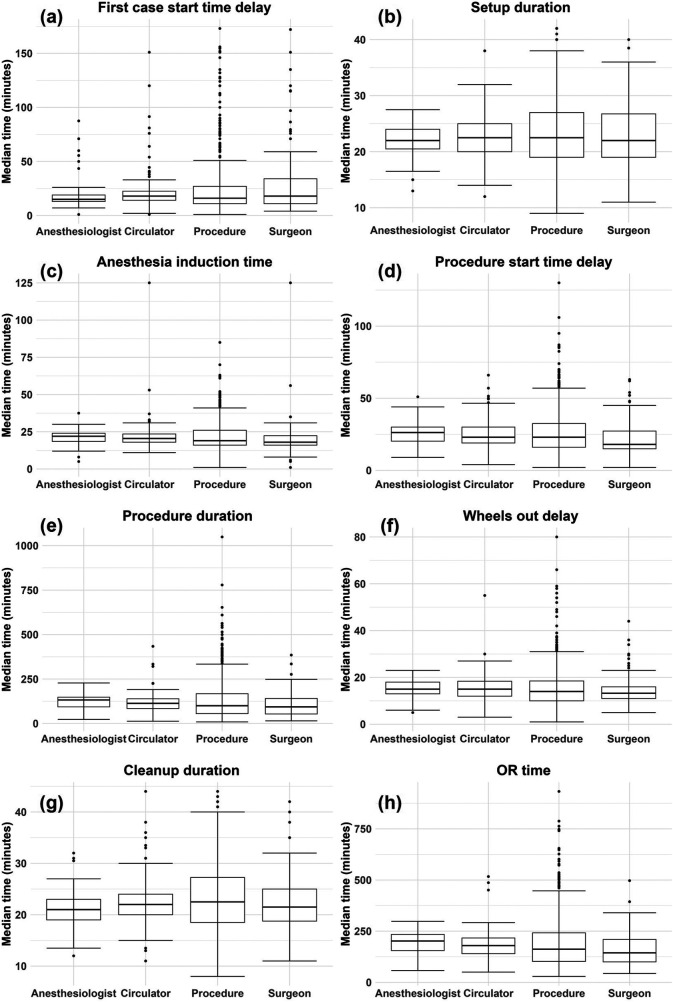
Box plots by random effect for each (untransformed) process time: **(A)** first case start time delay, **(B)** setup duration, **(C)** anesthesia induction time, **(D)** procedure start time delay, **(E)** procedure duration, **(F)** wheels out delay, **(G)** cleanup duration, **(H)** OR time. The values used to generate each box plot were the median times for each level of a given random effect and for each process time.

Figure 3 shows a summary of the data, after cleaning, for each (untransformed) process time and random effect. To calculate the values of a given box plot, the times of the corresponding process time (e.g., procedure duration) were grouped according to the levels of the corresponding random effect (e.g., surgeon) and the median was taken for each level. For all process times, the random effect of procedure displays the highest dispersion through larger interquartile ranges and wider outliers (dots). As seen in Table 2, procedure also has significantly more levels than any other random effect. However, this alone does not explain the higher dispersion observed in procedure. Rather, it is likely that procedure is an important factor, which also agrees with much of the literature concluding that procedure is the primary source of variability in various OR process times (11, 12, 16, 35). Figure 3 also shows that all process times are right-skewed, as indicated by the median line being closer to the first quartile (bottom of box) and the upper tails and outliers extending far upwards, particularly in the box plots corresponding to the random effect of procedure. Some process times only show mild skewness, such as cleanup duration.^14^

Table 4 and Supplementary Tables S2–S8 provide an assessment of the individual impact of each random effect, with and without fixed effects. In Table 4 and Supplementary Tables S2–S8, ${\text{ICC}}_{\left(\text{adj}\right)}$ shows a small reduction when fixed effects are included; thus, the fixed effects included in this study do not explain much variation in the logarithms of the process times. Of all main random effects, procedure shows the largest ${\text{ICC}}_{\left(\text{adj}\right)}$ for most of the process times. This observation is supported by Figure 3 in that the box plots associated with procedure show the largest variation. Exceptions include first case start time delay (Supplementary Table S2) and cleanup duration (Supplementary Table S7) in which surgeon shows the largest ${\text{ICC}}_{\left(\text{adj}\right)}$.^15^ In other cases, surgeon is not far behind procedure in terms of ${\text{ICC}}_{\left(\text{adj}\right)}$, including setup duration (Supplementary Table S3), anesthesia induction time (Supplementary Table S4), procedure start time delay (Supplementary Table S5), and wheels out delay (Supplementary Table S6). Procedure duration (Table 4) and OR time (Supplementary Table S8) are the process times where procedure explains moderately more variation than surgeon.^16^ While the fact that procedure and surgeon accounting for the most variability could in part be due to both factors having many levels, circulator also shows approximately the same number of levels as surgeon (Table 2), yet it typically accounted for very little of the variability. One final observation from Table 4 and Supplementary Tables S2–S8 is that the interaction terms typically have higher ${\text{ICC}}_{\left(\text{adj}\right)}$ than their main effect counterparts, but the gain is marginal.

**Table 4:** Univariate assessment of random effects when using procedure duration as the response.

Random effect	${\text{ICC}}_{\text{( adj) }}$ (%), without FE	${\text{ICC}}_{\text{( adj) }}$ (%), with FE
Procedure	66.9	59.8
Surgeon	39.7	34.9
Procedure × Surgeon	72.0	64.3
Anesthesiologist	6.1	1.6
Procedure × Anesthesiologist	69.8	58.2
Surgeon × Anesthesiologist	46.9	35.3
Circulator	12.3	6.6
Procedure × Circulator	69.0	59.1
Surgeon × Circulator	44.3	35.7
Anesthesiologist × Circulator	22.7	12.3

Each row corresponds to an LMM with a fixed intercept and the single random effect specified in column 1. Columns 2 and 3 give ${\text{ICC}}_{\text{( adj) }}$ values (Equation 1) for each univariate random effect model, both excluding (column 2) and including (column 3) all fixed effects in the LMM. ICC, intraclass correlation coefficient; FE, fixed effects.

Model selection was performed for each process time as described in Section 3.2. The model selection process is illustrated with procedure duration (Table 5).^17^ The p-values were determined from performing chi-squared tests between each model and its previous (nested) model in Table 5, and they were used to determine whether to retain a particular random effect for the final LMM. In the case of procedure duration (and OR time; Supplementary Table S15), all main effects and interactions were determined to be significant and were retained for the final model. The gain in AIC exhibited by every random effect in addition to procedure indicates that including each term will likely improve prediction. The number of final models in which each random effect appeared is shown in Table 6. Procedure was by default included in every model, but surgeon, circulator, and the interaction of procedure and circulator also appeared in every model. Anesthesiologist appeared in all models except for that of first case start time delay.

**Table 5:** Model selection for choosing random effects in the LMM where procedure duration is the response.

Model	AIC	AIC gain	p-value
Base model	22516.8	–	–
+ Surgeon	21955.9	560.9	<0.001
+ Procedure × Surgeon	21750.2	766.6	<0.001
+ Anesthesiologist	21685.7	831.0	<0.001
+ Procedure × Anesthesiologist	21681.0	835.7	0.010
+ Surgeon × Anesthesiologist	21669.3	847.5	<0.001
+ Circulator	21628.8	888.0	<0.001
+ Procedure × Circulator	21597.9	918.9	<0.001
+ Surgeon × Circulator	21584.8	932.0	<0.001
+ Anesthesiologist × Circulator	21578.2	938.6	0.003

The base model is given in Equation 2 and consists of a fixed intercept, all six fixed effects, and procedure as a random intercept. Additions appearing in this table are cumulative in the sense that each subsequent random effect was added to the model in the preceding row. AIC gain is the improvement in AIC from adding additional random effects onto the base model (calculated as AIC of the base model minus AIC of the larger model). AIC, Akaike information criterion.

**Table 6:** Number of final models in which each random and fixed effect appeared.

Random effect	Frequency	Fixed effect	Frequency
Procedure	8	Number of procedures	6
Surgeon	8	Number of panels	1
Anesthesiologist	7	Procedure level	4
Circulator	8	Cancer/noncancer	1
Procedure × Surgeon	5	Position	1
Procedure × Anesthesiologist	5	Patient class	8
Procedure × Circulator	8		
Surgeon × Anesthesiologist	4		
Surgeon × Circulator	4		
Anesthesiologist × Circulator	2		

A new model for each process time was formed by augmenting the base model (Equation 2) with the significant random effects shown in Table 5 and Supplementary Tables S9–S15. Then the individual impact of each fixed effect on the performance of the augmented LMM was assessed (refer to Section 3.2). Table 7 shows the performance associated with the fixed effects for procedure duration.^18^ Based on the p-values, the fixed effects retained for the final model for procedure duration were the number of procedures, procedure level, and patient class. Table 6 shows the number of final models for which each fixed effect was retained. Patient class was found significant for every process time and the number of procedures was found significant for all process times except first case start time delay (Supplementary Table S16) and cleanup duration (Supplementary Table S21).

**Table 7:** Model selection for choosing fixed effects in the LMM where procedure duration is the response.

Model	AIC	AIC loss	p-value
Base model	21578.2	–	–
BM − Number of procedures	22772.2	1194.1	<0.001
BM − Number of panels	21577.0	−1.2	0.362
BM − Procedure level	21665.5	87.4	<0.001
BM − Cancer/noncancer	21576.8	−1.3	0.413
BM − Position	21574.3	−3.9	0.928
BM − Patient class	21696.0	117.8	<0.001

The base model consists of a fixed intercept, all six fixed effects, and the random effects found to be significant from Table 5. Each fixed effect was removed from the base model, and each reduced model was compared to the base model via a chi-squared test. If the base model was found significant compared to the reduced model, then the corresponding fixed effect was retained. Subtractions appearing in this table are not cumulative and denote that only the indicated fixed effect was removed from the base model and all other fixed effects were included. AIC loss is the increase in AIC from removing a fixed effect from the base model (calculated as AIC of the reduced model minus AIC of the base model). AIC, Akaike information criterion; BM, base model.

${\text{ICC}}_{\text{( adj) }}$ (Equation 1) was calculated for each random effect appearing in each final model, and model ICC values, ${\text{ICC}}_{\text{LMM}}$ and ${\text{ICC}}_{\text{LMM( adj) }}$ (Equation 3), were also calculated for each process time (Table 8). From Table 8, it is observed that surgeon is the random effect with the highest ${\text{ICC}}_{\text{( adj) }}$ value for five of the process times, including first case start time delay, setup duration, procedure start time delay, wheels out delay, and cleanup duration. However, the highest ${\text{ICC}}_{\text{( adj) }}$ value surgeon obtains is 21.1% for procedure start time delay. Procedure has the highest ${\text{ICC}}_{\text{( adj) }}$ value for all other process times, including anesthesia induction time, procedure duration, and OR time. Procedure accounted for 44.2% and 45.5% of variability in the logarithm of procedure duration and OR time, respectively. For all other process times, procedure accounted for approximately 11% of variation or less. While anesthesiologist was found significant for seven process times, it accounted for at most 1.1% of variation (wheels out delay). Interestingly, circulator was found significant for all models and accounted for as much as 3.4% of variation (wheels out delay). However, both anesthesiologist and circulator do not individually account for much variation.

**Table 8:** ${\text{ICC}}_{\text{( adj) }}$ values for each random effect (Equation 1) appearing in each final model.

		FCSTD	SD	AIT	PSTD	PD	WOD	CD	ORT
${\text{ICC}}_{\text{( adj) }}$ (%)	Procedure	1.0	9.9	**11.4**	10.7	**44.2**	5.8	1.3	**45.5**
	Surgeon	**7.1**	**12.2**	7.3	**21.1**	10.8	**10.9**	**11.3**	13.3
	Anesthesiologist	-	0.8	0.8	0.4	0.2	1.1	0.5	0.3
	Circulator	0.2	2.3	0.7	0.7	0.5	3.4	1.5	0.4
	Proc. × Surg.	–	–	–	3.5	8.7	2.2	2.5	7.2
	Proc. × Anes.	–	–	–	0.9	0.3	1.1	2.7	0.6
	Proc. × Circ.	3.4	0.4	2.1	5.6	1.3	1.4	0.4	0.6
	Surg. × Anes.	–	–	–	0.7	0.3	0.7	-	0.7
	Surg. × Circ.	–	6.5	–	–	0.9	–	8.6	0.9
	Anes. × Circ.	–	–	–	–	0.3	–	–	0.2
${\text{ICC}}_{\text{LMM( adj) }}$ (%)		11.6	32.1	22.3	43.5	67.5	26.5	28.7	69.7
${\text{ICC}}_{\text{LMM}}$ (%)		11.3	29.8	21.1	42.2	58.2	25.0	28.2	59.0

A dash (–) indicates the random effect was not selected for the final model. The random effects with the largest ICC for each process time are indicated in bold. Also provided are the model ICCs, namely ${\text{ICC}}_{\text{LMM}}$ and ${\text{ICC}}_{\text{LMM( adj) }}$ (Equation 3). FCSTD, First case start time delay; SD, Setup duration; AIT, Anesthesia induction time; PSTD, Procedure start time delay; PD, Procedure duration; WOD, Wheels out delay; CD, Cleanup duration; ORT, OR time.

Table 8 also shows several significant interaction terms. In particular, the interaction of procedure and circulator was significant for all models. In many cases, this interaction term accounted for more variation than circulator individually. This suggests that the effect of the primary circulating nurse is significant but their effect can depend on the type of procedure. In addition, the interaction of procedure and surgeon was significant for five models and accounted for 2.2%–8.7% of variation. This also suggests the effect of the surgeon depends on the procedure. Lastly, the interaction of surgeon and circulator accounted for a modest amount of variance in the logarithms of setup duration (6.5%) and cleanup duration (8.6%), suggesting a synergistic effect of surgical teams in some stages of the OR process.

Overall, Table 8 shows that the primary factors examined in this study - procedure type, primary surgeon, responsible anesthesia provider, and primary circulating nurse - are most impactful on procedure duration and OR time, accounting for 67.5% and 69.7% of variation (in the logarithms), respectively, after fixed effects have been accounted for. The primary factors also explained a moderate amount of variation in the logarithm of procedure start time delay (43.5%), and were mildly impactful on setup duration (32.1%), anesthesia induction time (22.3%), wheels out delay (26.5%), and cleanup duration (28.7%). The primary factors accounted for very little of the variation in the logarithm of first case start time delay (11.6%). Finally, it is noted that there were little differences between ${\text{ICC}}_{\text{LMM( adj) }}$ and ${\text{ICC}}_{\text{LMM}}$, further reinforcing that the fixed effects included in this study had little impact on the process times.

## 5 Discussion

### 5.1 Primary findings

The present study made several findings that both complement and add to the existing literature on OR efficiency. First, this study shows that, when investigating the impact of factors on the OR process, a fine-grained approach is necessary to pinpoint where in the process, and by how much, each factor makes an impact. In Section 1, it was hypothesized that the fine-grained stages of the OR process do not consist of the same sources of variability, nor that the common sources of variability account for the same proportion of variance in each stage. The results of this study support the above hypotheses (Table 8). Notably, OR time is an aggregate quantity consisting of the stages of the OR process in which the patient is present in the OR (i.e., “wheels in” to “wheels out”). However, the results of this study indicate that the quantification of variability in OR time mainly reflects the quantification of variability in procedure duration. Comparing the two process times in Table 8, their variabilities roughly decompose in the same way. For example, procedure accounted for 45.5% and 44.2% and surgeon for 13.3% and 10.8% of variability in the logarithms of OR time and procedure duration, respectively. Moreover, the random effects overall accounted for 69.7% and 67.5% of variability in the logarithms of OR time and procedure duration, respectively. In addition to procedure duration, OR time also comprises the time intervals associated with (a large proportion of) anesthesia induction time, procedure start time delay, and wheels out delay. However, the decompositions of variability for the latter three process times bear little resemblance to that of OR time. Thus, what happens in the OR during the procedure is mostly what is driving the aggregate quantity of OR time. As a result, interventions for improving efficiency in OR time should be focused on the procedure stage.

The second primary finding regards the impacts of each human factor. In particular, the primary surgeon had a larger impact in this study than what was previously reported in the literature. For instance, van Eijk et al. (12) found that the primary surgeon and second surgeon^19^ only accounted for a combined 4.8% of the variability in the logarithm of OR time. In the present study, however, primary surgeon alone accounted for 13.3% of variability in the logarithm of OR time (Table 8). Surgeon also accounted for a substantial 21.1% of variability in the logarithm of procedure start time delay and for at least 7% in the logarithms of all other process times (Table 8). The above results suggest that the primary surgeon (and other surgeons in the team) have moderate impacts not only on procedure duration, but also on many stages of the OR process. The importance of the surgeon was stressed in previous literature, e.g., Strum et al. (16), however a quantification of the variability due to surgeon was usually not provided. Moreover, the impact of the surgeon depends in part on the procedure, as seen by the significant interaction term of procedure and surgeon (Table 8). Indeed, Strum et al. (16) found that variability in surgical time increased as procedure time increased, indicating an interaction effect between type of procedure and surgeon.

In agreement with previous literature, responsible anesthesia provider was often a significant factor but not impactful on OR efficiency (12, 16). Surprisingly, responsible anesthesia provider had little impact on the anesthesia-controlled times, including anesthesia induction time and wheels out delay, the latter of which includes the patient’s emergence from anesthesia. Other factors not included in this study, such as patient and operation characteristics, may be important for accounting for variability in anesthesia-controlled times (54).

Lastly, this study found the primary circulating nurse, a less studied human factor in the literature regarding OR efficiency, to be a significant factor in all stages of the OR process. This is reasonable because the circulating nurse, sometimes called the “perioperative” nurse, is involved before the surgery (e.g., transporting the patient and preparing the patient for surgery), during the surgery (e.g., assisting with equipment), and after the surgery (e.g., monitoring the patient) (55). In this study, the circulating nurse had their largest effect on wheels out delay and setup duration, accounting for 3.4% and 2.3% of variability (Table 8), respectively. In addition, the interaction of procedure and circulator was also significant for every process time, and the interaction of surgeon and circulator was significant for four process times and reached an ${\text{ICC}}_{\text{( adj) }}$ value as high as 8.6% (cleanup duration; Table 8). Thus, the effect of the circulating nurse depends on the procedure type and, for some stages of the OR process, also on the particular attending surgeon, indicating some team synergistic effect on OR efficiency. Indeed, studies have found that nursing staff characteristics and team effects are important components of OR efficiency (56–58). More work is needed though to investigate the role of nursing staff on OR efficiency and to design interventions with nursing staff as a central component.

### 5.2 Clinical implications

The primary findings of this paper have the following clinical implications. First, OR process prediction models may be improved by incorporating significant factors and interactions found in this study. Improving prediction models will improve scheduling accuracy and increase OR utilization (i.e., decrease under- and over-utilization) which directly impacts OR efficiency.^20^ This paper helps to fill a gap by quantifying the effect of key members of the surgical team and procedure type on various stages of the OR process. For instance, it may be beneficial for models predicting procedure duration to not only include the procedure type and primary surgeon, but also consider their interaction (Table 8). There is likely less variability in procedure duration among surgeons for simple procedures than for more complex procedures. Thus, prediction models should take into account that a surgeon’s variability itself will vary depending on the type of procedure performed. In addition, this study uniquely identifies the primary circulating nurse and various interaction terms as significant; therefore, researchers can more comprehensively consider the members of surgical teams and their synergistic effects when designing prediction models.

Second, case scheduling may also be improved by incorporating significant factors and interactions found in this study. The effect of a particular individual, e.g., the attending primary surgeon, can be considered when allocating portions of time to each stage for a given case. The particular individual can be used in a more advanced prediction model as mentioned above or, more simply, the individual’s historical data can be considered when allocating times. The same process can be done regarding surgical teams or combinations of surgical team members. Also, knowledge of particular surgical team members and teams themselves can inform strategies for case scheduling. For instance, if a particular surgeon or surgical team is known to have higher variability or expected completion times for a particular case, then such a case could be scheduled earlier or first in the day to allow for dynamic scheduling after the case’s completion, which could allow for the completion of more cases in a day (16).

Third, OR efficiency can be improved by minimizing the variability in the stages of the OR process attributable to members of the surgical team and combinations of team members. The present study highlights areas of higher variability for surgical team members. Efforts could be made to reduce variability by identifying inefficiencies in a surgical team’s or team member’s practice and providing relevant training. If the area of improvement is in teamwork, for instance, training could seek to promote effective, assertive, and closed-loop communication among surgical teams to help minimize team performance variability.^21^ Moreover, surgical teams can be further streamlined to match surgeons, anesthesiologists, and nurses who consistently work well together, which will in turn reduce performance variability.

### 5.3 Study limitations and future work

**Figure 4:**
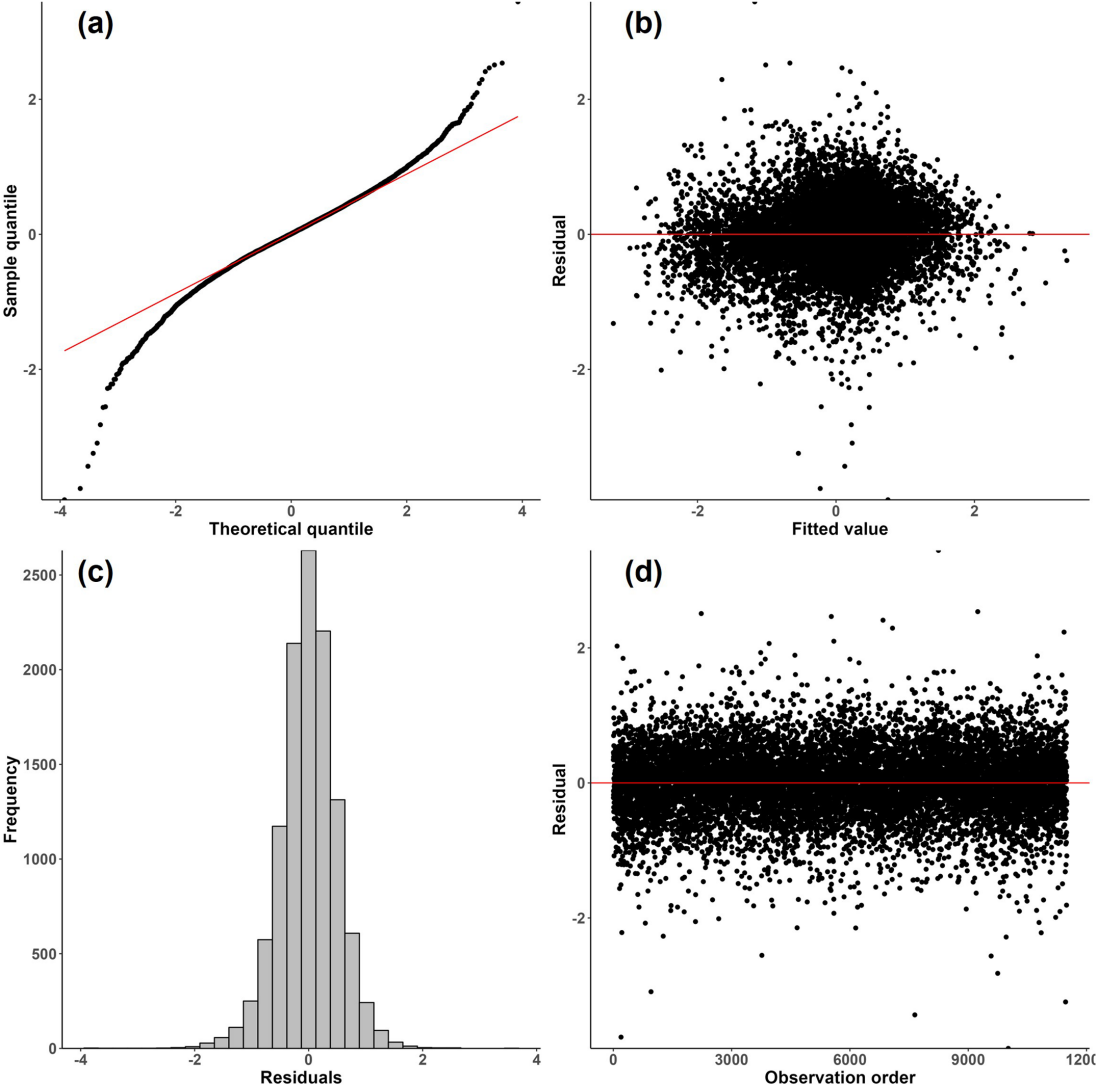
Diagnostic plots for the final LMM where procedure duration is the response. **(a)** Normal probability plot of residuals; **(b)** residuals vs. fitted values; **(c)** histogram of residuals; **(d)** residuals vs. observation order.

A limitation of this work was the use of a linear modeling approach and transformations to conform to model assumptions. Figure 4 shows model diagnostic plots for procedure duration.^22^ Figure 4(a) shows some departure from a normal distribution for the logarithm of procedure duration; the distribution shows heavier tails as indicated by the upper right and lower left portions of the curve “peeling away” from the red line. Heavier tails indicate the presence of outliers in both directions. In addition, Figure 4(b) suggests mild heteroscedasticity in the residuals as the variation appears to decrease as fitted values increase in absolute value. Finally, the distribution of procedure duration exhibited right skewness, which was corrected by a log transformation. The above three observations were true for many of the process times. While the results provided in this paper are still relatively robust due to the large sample size of the dataset, more accurate results could possibly be obtained through the use of robust regression methods suited to handle outliers and heteroscedasticity. Moreover, generalized linear mixed models could be explored to handle the non-normality of the process times (59).

Another limitation of this work was the lack of inclusion of many potentially important fixed-effect variables. Previous literature has explored a wide range of factors that may contribute to OR efficiency (refer to Section 2). There are likely important factors missing from this analysis as they were not available in the database at the University of Miami hospital. Future work could explore a more comprehensive list of factors to maximize the potential of data to reveal OR inefficiencies. Moreover, even with a more comprehensive and retrospective assessment of influential factors, more proactive measures are needed that implement realistic interventions, in collaboration with members of surgical teams, to bring greater efficiency to the OR suite.

## 6 Conclusions

The primary goal of this paper was to quantify the extent to which the procedure type and key members of the surgical team accounted for variation in the fine-grained stages of the OR process. Some of the stages of the OR process and more aggregate process times have been analyzed previously in the literature (refer to Section 2.1). However, a comprehensive analysis of the impact of the primary surgical team members on the many stages comprising the OR process is lacking. This study helps to fill this gap by developing eight different linear mixed models that quantify the variability of several OR process times with respect to procedure type, primary surgeon, responsible anesthesiology provider, and primary circulating nurse.

This study found that, to more accurately account for sources of variability in the OR process, it is necessary to break up the OR process into smaller, homogeneous stages. For instance, this study found that OR time, defined as the “wheels in” to “wheels out” time of a patient in the OR, largely reflects procedure duration and is therefore not homogeneous across its entire time span. In addition, this study found that surgeon has a larger impact than previously reported in the literature and that the circulating nurse accounted for a significant, albeit small, proportion of variability in all eight process times studied. This study can serve as a foundation for quantifying the impact of important members of the surgical team on various stages of the OR process and for more targeted interventions seeking to realize more efficient and cost-effective OR suites.

## Data Availability

The datasets presented in this article are not readily available because of concerns regarding data privacy. Requests to access the datasets should be directed to Adam Meyers, axm8336@miami.edu.

## Footnotes

^1^For comprehensive reviews, refer to Lee et al. (7) and Dexter and Epstein (8).

^2^ACT is defined in Dexter et al. (22) as the sum of the time from when the patient enters the OR to when the positioning or skin preparation begins, plus the time from when the surgical dressing is completed to when the patient is wheeled out of the OR.

^3^The “golden patient” initiative is a strategy where the first patient on the operating list is medically fit, thoroughly investigated, and has a clear surgical plan (25).

^4^The “productive operating theatre” scheme is a three-step intervention to increase OR efficiency (25, 26).

^5^Surgical-controlled time is defined as the duration of time from surgical incision to surgical closure.

^6^Further description of the dataset in terms of procedural categories and the range of CPT codes used are provided in Supplementary Table S1.

^7^Initially, the process times of anesthesia start time delay, computed as anesthesia start minus wheels in, and next case start time delay, computed as setup start (of next case) minus cleanup end (of previous case), were included in this study. However, after data cleaning, there were too little data to build LMMs with the desired factors; thus these process times were excluded from the analysis.

^8^Supplementary Figures S1–S6 show the corresponding histograms for all other process times.

^9^However, most cases involved only a few procedures.

^10^All LMMs were fitted using the **lmer** function from the R package **lme4** (53). A reference on this package is provided by Bates et al. (53).

^11^A similar model selection procedure to that of van Eijk et al. (12) was used in this study.

^12^Because all fixed effects were categorical with many levels, several dummy variables were created which would be included in the vector $X_{i}$.

^13^The standard 0.05 level of significance was used.

^14^Skewness of the process times is further supported by the histograms displayed in Figure 2 and Supplementary Figures S1–S6.

^15^For cleanup duration (Supplementary Table S7), surgeon is only higher than procedure in ${\text{ICC}}_{\left(\text{adj}\right)}$ when fixed effects are included.

^16^As concluded in this paper, though, the variability of OR time largely reflects that of procedure duration; thus, procedure type has a significantly higher impact on procedure duration than the primary surgeon, but this is not the case for the other stages of the OR process as examined in this study.

^17^The corresponding tables for all other process times are included in the Supplementary Tables S9–S15.

^18^Supplementary Tables S16–S22 show the performance associated with the fixed effects for all other process times.

^19^“Second surgeon” is defined in van Eijk et al. (12) as the first registered assistant surgeon during a procedure.

^20^See Dexter and Epstein (8) where “OR efficiency” is defined as minimizing the “inefficiency of use of OR time,” the latter of which is calculated using costs and times associated with under- and over-utilization.

^21^Granted, targeted research is needed to identify areas of inefficiency.

^22^Diagnostic plots for all other process times are provided in Supplementary Figures S7–S13.
