# Supplementary material for: Quantifying the impact of surgical teams on each stage of the operating room process
Source: Front Digit Health. 2024 Oct 3;6:1455477. doi: 10.3389/fdgth.2024.1455477 (PMC11484065; doi:10.3389/fdgth.2024.1455477)
Supplement: Supplementary file 1 [file Datasheet1.pdf]

Supplementary Material

This document contains the following supplementary materials:

- Tab. S1: Description of dataset used including CPT codes;
- Fig. S1-S6: Histograms of the responses before and after transformation;
- Tab. S2-S8: Univariate assessment of random effects;
- Tab. S9-S15: Model selection for choosing random effects;
- Tab. S16-S22: Model selection for choosing fixed effects;
- Fig. S7-S13: Diagnostic plots for final linear mixed models.

Table S1. Description of database after data cleaning was performed common to all process times (e.g., removal of weekend days). Eighteen descriptors are used to categorize the procedures, and each category's most frequently occurring procedure is listed. Abbreviations: C, number of cases; P, number of procedures (i.e., number of unique CPT codes); S, number of surgeons; A, number of anesthesiologists; N, number of circulator nurses.

Descriptor	C	P	S	A	N	Most Frequent Procedure & CPT code		Range of CPT Codes	
Anesthesia Procedures	104	7	11	36	36	Surgical Laparoscopy with Cholecystectomy	540	100	1274
Medicine Services and Procedures	16	5	12	12	11	Tissue Debridement - Total Wound(s) Surface Area; First 20 sq cm or less	97597	92502	97607
Operating Microscope Procedures	8	1	7	7	5	Microsurgical Techniques, Requiring Use of Operating Microscope	69990	69990	69990
Radiology Procedures	3	1	1	3	3	Diagnostic Radiology (Imaging of Veins and Lymphatics)	75801	70010	77779
Maternity Care and Delivery	3	3	1	3	3	Induced Abortion, by Dilation and Evacuation	59841	5900	59899
Auditory System	4	3	3	3	4	Excision External Ear; Partial, Simple Repair	69110	6900	69979
Cardiovascular System	273	41	24	48	60	Endovenous Ablation Therapy of Incompetent Vein, Laser	36478	33016	37799
Digestive System	660	116	44	61	84	Anorectal Exam, Surgical, Requiring Anesthesia	45990	40490	49999
Endocrine System	20	6	6	15	13	Thyroidectomy, Total or Complete	60240	60000	60699
Eye and Ocular Adnexa	17	4	9	12	14	Revision or Repair of Operative Wound of Anterior Segment	66250	65091	68899
Female Genital System	484	59	16	58	59	Hysteroscopy, Surgical with Sampling or Polypectomy	58558	56405	58999
Hemic and Lymphatic Systems	45	7	8	19	22	Injection Procedure; Lymphangiography	38790	38100	38999
Integumentary System	386	93	62	57	83	Incision and Drainage, Complex Postoperative Wound Infection	10180	10030	19499
Male Genital System	4	2	4	4	4	Scrotal Exploration	55110	54000	55889
Musculoskeletal System	2492	226	58	72	89	Arthrodesis, Anterior Interbody, Cervical Below C2	22551	20100	29999
Nervous System	6252	196	38	74	100	Craniectomy, Bone Flap Craniotomy for Excision of Brain Tumor	61510	61000	64999
Respiratory System	721	47	27	65	69	Nasal/Sinus Endoscopy with Ethmoidectomy	31259	30000	32999
Urinary System	25	10	9	13	12	Cystourethroscopy with Insertion of Ureteral Stent	53332	50010	53899

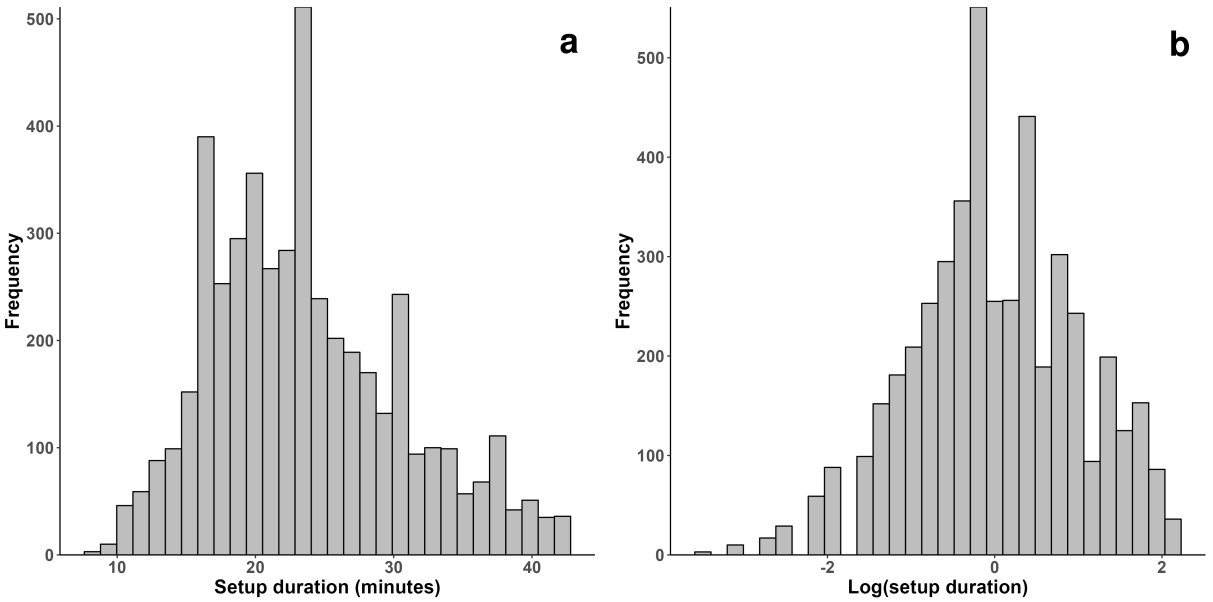

Figure S1: Histograms for setup duration (a) before transformation and (b) after a log transformation.

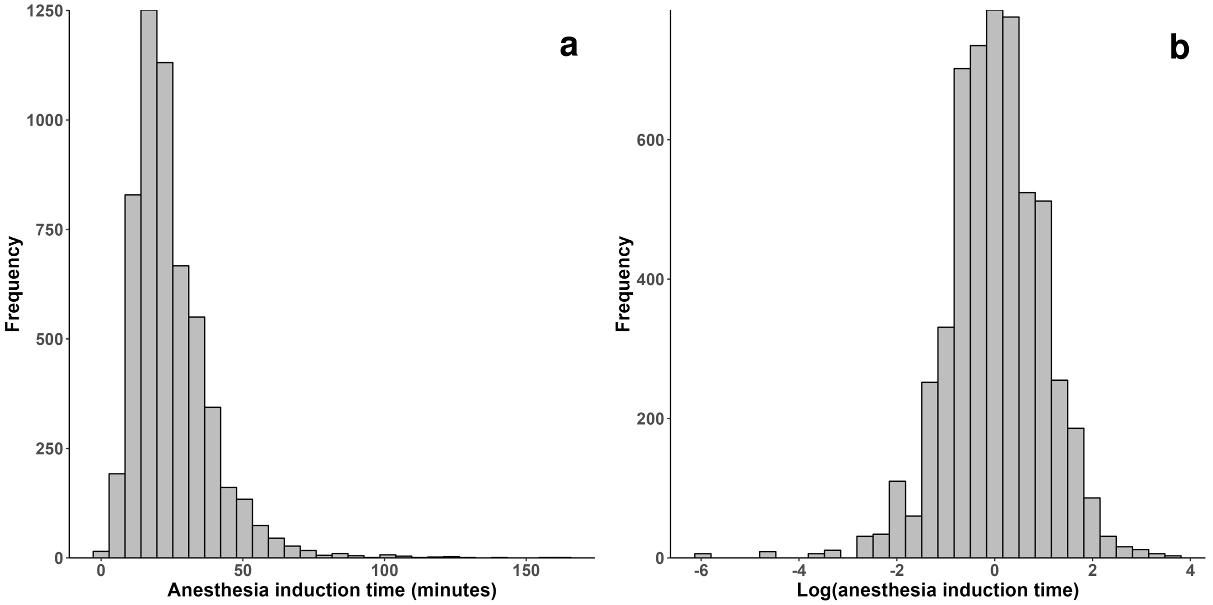

Figure S2: Histograms for anesthesia induction time (a) before transformation and (b) after a log transformation.

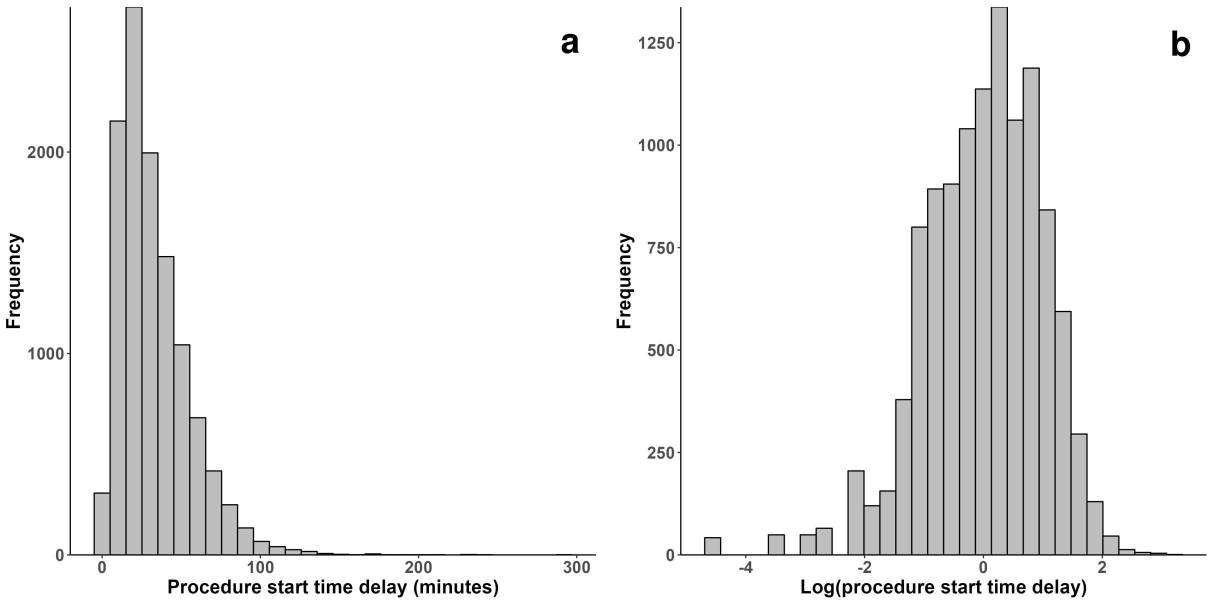

Figure S3: Histograms for procedure start time delay (a) before transformation and (b) after a log transformation.

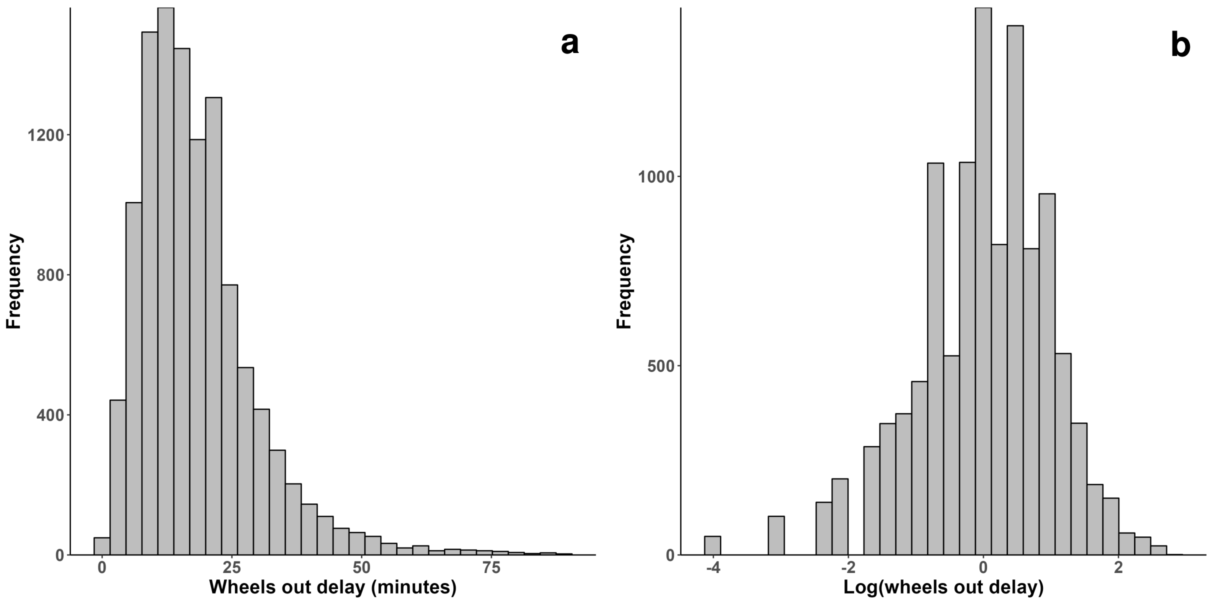

Figure S4: Histograms for wheels out delay (a) before transformation and (b) after a log transformation.

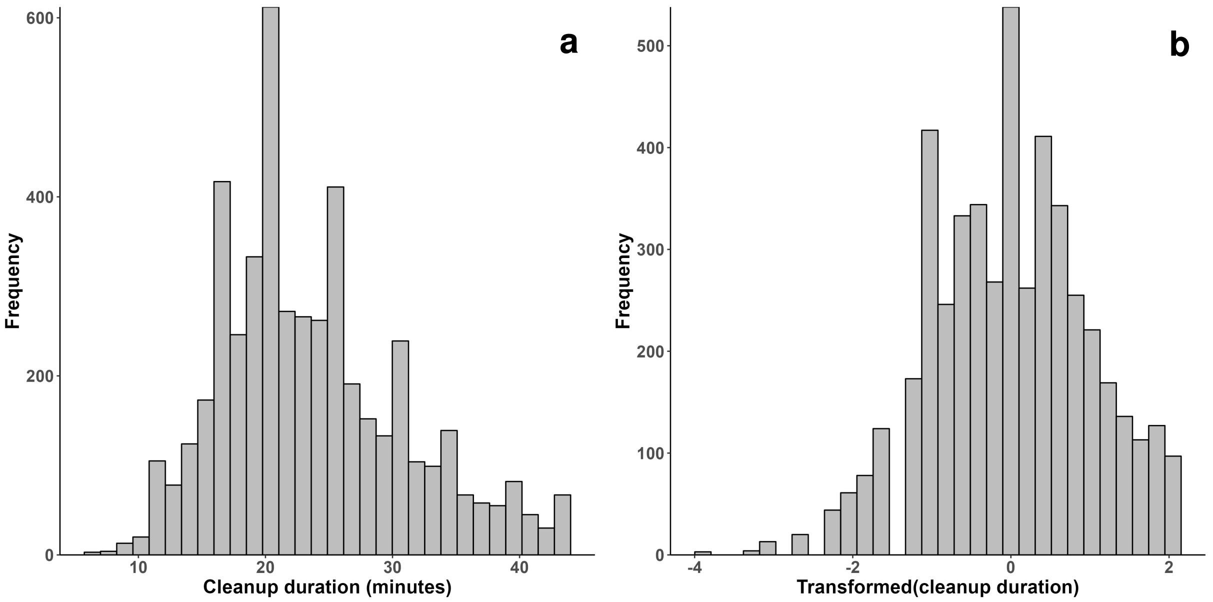

Figure S5: Histograms for cleanup duration (a) before transformation and (b) after a log transformation.

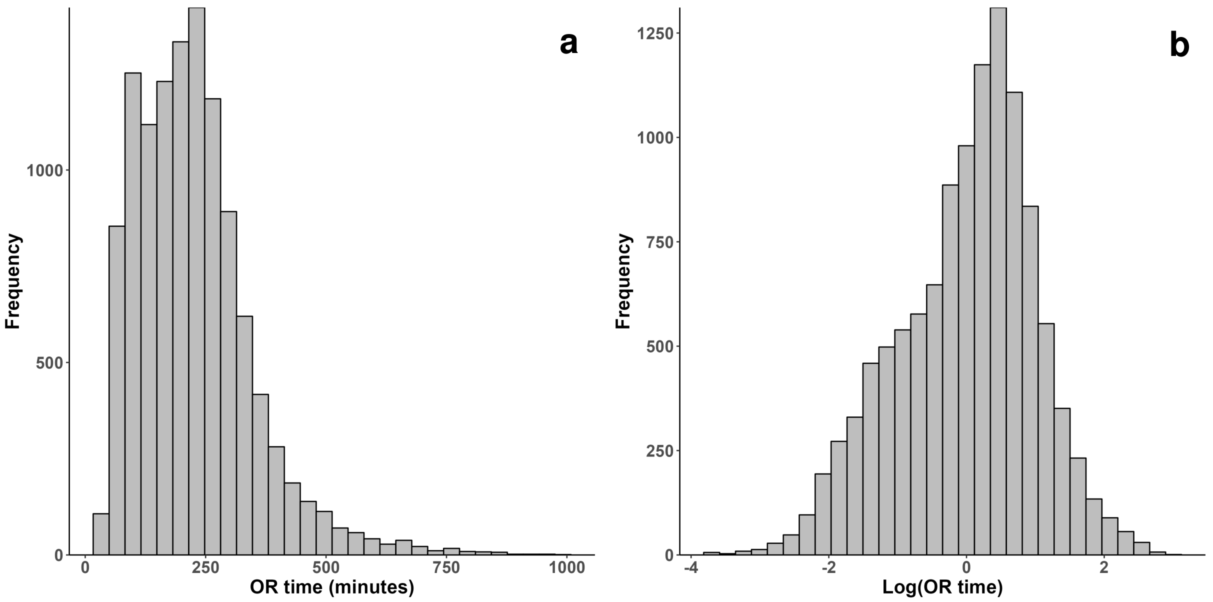

Figure S6: Histograms for OR time (a) before transformation and (b) after a log transformation.

Table S2. Univariate assessment of random effects when using first case start time delay as the response. Each row corresponds to an LMM with a fixed intercept and the single random effect specified in column 1. Columns 2 and 3 give $ICC_{(adj)}$ values (Eq. (1) in the main paper) for each univariate random effect model, both excluding (column 2) and including (column 3) all fixed effects in the LMM. Abbreviations: ICC, intraclass correlation coefficient; FE, fixed effects.

Random effect	$ICC_{(adj)}$ (%), without FE	$ICC_{(adj)}$ (%), with FE
Procedure	8.0	6.5
Surgeon	9.5	8.4
Procedure × Surgeon	11.6	9.6
Anesthesiologist	0.2	0.1
Procedure × Anesthesiologist	8.7	5.3
Surgeon × Anesthesiologist	11.0	9.8
Circulator	0.7	0.9
Procedure × Circulator	9.7	7.8
Surgeon × Circulator	8.0	7.0
Anesthesiologist × Circulator	1.7	1.8

Table S3. Univariate assessment of random effects when using setup duration as the response. Each row corresponds to an LMM with a fixed intercept and the single random effect specified in column 1. Columns 2 and 3 give $ICC_{(adj)}$ values (Eq. (1) in the main paper) for each univariate random effect model, both excluding (column 2) and including (column 3) all fixed effects in the LMM. Abbreviations: ICC, intraclass correlation coefficient; FE, fixed effects.

Random effect	$ICC_{(adj)}$ (%), without FE	$ICC_{(adj)}$ (%), with FE
Procedure	34.0	20.5
Surgeon	28.4	19.2
Procedure × Surgeon	38.0	22.8
Anesthesiologist	7.1	3.5
Procedure × Anesthesiologist	39.6	17.4
Surgeon × Anesthesiologist	35.6	18.6
Circulator	13.6	7.6
Procedure × Circulator	43.0	24.2
Surgeon × Circulator	40.1	26.0
Anesthesiologist × Circulator	20.2	7.2

Table S4. Univariate assessment of random effects when using anesthesia induction time as the response. Each row corresponds to an LMM with a fixed intercept and the single random effect specified in column 1. Columns 2 and 3 give $ICC_{(adj)}$ values (Eq. (1) in the main paper) for each univariate random effect model, both excluding (column 2) and including (column 3) all fixed effects in the LMM. Abbreviations: ICC, intraclass correlation coefficient; FE, fixed effects.

Random effect	$ICC_{(adj)}$ (%), without FE	$ICC_{(adj)}$ (%), with FE
Procedure	27.9	17.3
Surgeon	21.5	13.6
Procedure × Surgeon	33.0	20.5
Anesthesiologist	3.8	1.5
Procedure × Anesthesiologist	31.6	15.1
Surgeon × Anesthesiologist	24.1	10.8
Circulator	5.9	1.8
Procedure × Circulator	35.0	19.0
Surgeon × Circulator	23.1	10.3
Anesthesiologist × Circulator	8.5	3.3

Table S5. Univariate assessment of random effects when using procedure start time delay as the response. Each row corresponds to an LMM with a fixed intercept and the single random effect specified in column 1. Columns 2 and 3 give $ICC_{(adj)}$ values (Eq. (1) in the main paper) for each univariate random effect model, both excluding (column 2) and including (column 3) all fixed effects in the LMM. Abbreviations: ICC, intraclass correlation coefficient; FE, fixed effects.

Random effect	$ICC_{(adj)}$ (%), without FE	$ICC_{(adj)}$ (%), with FE
Procedure	37.7	30.4
Surgeon	31.2	29.7
Procedure × Surgeon	46.0	38.2
Anesthesiologist	5.1	2.2
Procedure × Anesthesiologist	44.5	32.4
Surgeon × Anesthesiologist	37.8	30.5
Circulator	8.6	5.0
Procedure × Circulator	47.6	36.3
Surgeon × Circulator	37.4	30.3
Anesthesiologist × Circulator	18.5	10.0

Table S6. Univariate assessment of random effects when using wheels out delay as the response. Each row corresponds to an LMM with a fixed intercept and the single random effect specified in column 1. Columns 2 and 3 give $ICC_{(adj)}$ values (Eq. (1) in the main paper) for each univariate random effect model, both excluding (column 2) and including (column 3) all fixed effects in the LMM. Abbreviations: ICC, intraclass correlation coefficient; FE, fixed effects.

Random effect	$ICC_{(adj)}$ (%), without FE	$ICC_{(adj)}$ (%), with FE
Procedure	29.8	18.1
Surgeon	20.5	15.0
Procedure × Surgeon	31.9	18.3
Anesthesiologist	5.8	2.2
Procedure × Anesthesiologist	33.5	16.5
Surgeon × Anesthesiologist	28.5	15.1
Circulator	10.2	5.7
Procedure × Circulator	33.9	17.7
Surgeon × Circulator	30.1	18.8
Anesthesiologist × Circulator	14.5	6.2

Table S7. Univariate assessment of random effects when using cleanup duration as the response. Each row corresponds to an LMM with a fixed intercept and the single random effect specified in column 1. Columns 2 and 3 give $ICC_{(adj)}$ values (Eq. (1) in the main paper) for each univariate random effect model, both excluding (column 2) and including (column 3) all fixed effects in the LMM. Abbreviations: ICC, intraclass correlation coefficient; FE, fixed effects.

Random effect	$ICC_{(adj)}$ (%), without FE	$ICC_{(adj)}$ (%), with FE
Procedure	20.7	14.4
Surgeon	17.8	14.6
Procedure × Surgeon	23.7	16.8
Anesthesiologist	9.6	6.0
Procedure × Anesthesiologist	29.7	18.1
Surgeon × Anesthesiologist	27.7	20.2
Circulator	12.3	7.5
Procedure × Circulator	31.9	21.6
Surgeon × Circulator	33.5	26.9
Anesthesiologist × Circulator	20.9	11.5

Table S8. Univariate assessment of random effects when using OR time as the response. Each row corresponds to an LMM with a fixed intercept and the single random effect specified in column 1. Columns 2 and 3 give $ICC_{(adj)}$ values (Eq. (1) in the main paper) for each univariate random effect model, both excluding (column 2) and including (column 3) all fixed effects in the LMM. Abbreviations: ICC, intraclass correlation coefficient; FE, fixed effects.

Random effect	$ICC_{(adj)}$ (%), without FE	$ICC_{(adj)}$ (%), with FE
Procedure	68.2	61.2
Surgeon	42.5	38.4
Procedure × Surgeon	74.5	66.9
Anesthesiologist	8.1	2.1
Procedure × Anesthesiologist	72.3	60.5
Surgeon × Anesthesiologist	52.7	41.7
Circulator	13.8	6.9
Procedure × Circulator	70.6	60.2
Surgeon × Circulator	49.5	41.0
Anesthesiologist × Circulator	25.4	12.6

Table S9. Model selection for choosing random effects in the LMM where first case start time delay is the response. The base model is given in Eq. (2) of the main paper and consists of a fixed intercept, all six fixed effects, and procedure as a random intercept. Additions appearing in this table are cumulative in the sense that each subsequent random effect was added to the model in the preceding row. AIC gain is the improvement in AIC from adding additional random effects onto the base model (calculated as AIC of the base model minus AIC of the larger model). Abbreviations: AIC, Akaike information criterion.

Model	AIC	AIC gain	p-value
Base model	9888.0	-	-
+ Surgeon	9846.2	41.8	<0.001
+ Procedure × Surgeon	9848.2	39.8	0.918
+ Anesthesiologist	9850.2	37.8	1.000
+ Procedure × Anesthesiologist	9852.2	35.8	1.000
+ Surgeon × Anesthesiologist	9852.4	35.6	0.182
+ Circulator	9848.9	39.1	0.019
+ Procedure × Circulator	9842.9	45.1	0.005
+ Surgeon × Circulator	9836.4	51.6	0.004
+ Anesthesiologist × Circulator	9838.4	49.6	0.999

Table S10. Model selection for choosing random effects in the LMM where setup duration is the response. The base model is given in Eq. (2) of the main paper and consists of a fixed intercept, all six fixed effects, and procedure as a random intercept. Additions appearing in this table are cumulative in the sense that each subsequent random effect was added to the model in the preceding row. AIC gain is the improvement in AIC from adding additional random effects onto the base model (calculated as AIC of the base model minus AIC of the larger model). Abbreviations: AIC, Akaike information criterion.

Model	AIC	AIC gain	p-value
Base model	11783.4	-	-
+ Surgeon	11685.5	98.0	<0.001
+ Procedure × Surgeon	11685.5	98.0	0.157
+ Anesthesiologist	11664.2	119.3	<0.001
+ Procedure × Anesthesiologist	11666.2	117.3	1.000
+ Surgeon × Anesthesiologist	11668.2	115.3	1.000
+ Circulator	11598.5	184.9	<0.001
+ Procedure × Circulator	11596.5	186.9	0.045
+ Surgeon × Circulator	11572.8	210.7	<0.001
+ Anesthesiologist × Circulator	11574.8	208.7	0.998

Table S11. Model selection for choosing random effects in the LMM where anesthesia induction time is the response. The base model is given in Eq. (2) of the main paper and consists of a fixed intercept, all six fixed effects, and procedure as a random intercept. Additions appearing in this table are cumulative in the sense that each subsequent random effect was added to the model in the preceding row. AIC gain is the improvement in AIC from adding additional random effects onto the base model (calculated as AIC of the base model minus AIC of the larger model). Abbreviations: AIC, Akaike information criterion.

Model	AIC	AIC gain	p-value
Base model	14062.1	-	-
+ Surgeon	13961.7	100.4	<0.001
+ Procedure × Surgeon	13963.7	98.4	1.000
+ Anesthesiologist	13931.6	130.5	<0.001
+ Procedure × Anesthesiologist	13933.6	128.5	1.000
+ Surgeon × Anesthesiologist	13934.7	127.4	0.330
+ Circulator	13921.0	141.1	<0.001
+ Procedure × Circulator	13917.8	144.4	0.022
+ Surgeon × Circulator	13919.5	142.6	0.623
+ Anesthesiologist × Circulator	13921.5	140.6	1.000

Table S12. Model selection for choosing random effects in the LMM where procedure start time delay is the response. The base model is given in Eq. (2) of the main paper and consists of a fixed intercept, all six fixed effects, and procedure as a random intercept. Additions appearing in this table are cumulative in the sense that each subsequent random effect was added to the model in the preceding row. AIC gain is the improvement in AIC from adding additional random effects onto the base model (calculated as AIC of the base model minus AIC of the larger model). Abbreviations: AIC, Akaike information criterion.

Model	AIC	AIC gain	p-value
Base model	26871.7	-	-
+ Surgeon	25746.7	1125.0	<0.001
+ Procedure × Surgeon	25693.6	1178.1	<0.001
+ Anesthesiologist	25638.4	1233.3	<0.001
+ Procedure × Anesthesiologist	25616.7	1255.0	<0.001
+ Surgeon × Anesthesiologist	25608.3	1263.4	0.001
+ Circulator	25513.5	1358.3	<0.001
+ Procedure × Circulator	25397.3	1474.5	<0.001
+ Surgeon × Circulator	25395.7	1476.0	0.060
+ Anesthesiologist × Circulator	25396.1	1475.6	0.203

Table S13. Model selection for choosing random effects in the LMM where wheels out delay is the response. The base model is given in Eq. (2) of the main paper and consists of a fixed intercept, all six fixed effects, and procedure as a random intercept. Additions appearing in this table are cumulative in the sense that each subsequent random effect was added to the model in the preceding row. AIC gain is the improvement in AIC from adding additional random effects onto the base model (calculated as AIC of the base model minus AIC of the larger model). Abbreviations: AIC, Akaike information criterion.

Model	AIC	AIC gain	p-value
Base model	28785.2	-	-
+ Surgeon	28569.5	215.7	<0.001
+ Procedure × Surgeon	28552.5	232.7	<0.001
+ Anesthesiologist	28425.1	360.0	<0.001
+ Procedure × Anesthesiologist	28409.9	375.3	<0.001
+ Surgeon × Anesthesiologist	28407.8	377.4	0.043
+ Circulator	28239.6	545.6	<0.001
+ Procedure × Circulator	28228.0	557.2	<0.001
+ Surgeon × Circulator	28228.5	556.7	0.223
+ Anesthesiologist × Circulator	28229.3	555.8	0.284

Table S14. Model selection for choosing random effects in the LMM where cleanup duration is the response. The base model is given in Eq. (2) of the main paper and consists of a fixed intercept, all six fixed effects, and procedure as a random intercept. Additions appearing in this table are cumulative in the sense that each subsequent random effect was added to the model in the preceding row. AIC gain is the improvement in AIC from adding additional random effects onto the base model (calculated as AIC of the base model minus AIC of the larger model). Abbreviations: AIC, Akaike information criterion.

Model	AIC	AIC gain	p-value
Base model	12879.1	-	-
+ Surgeon	12783.0	96.0	<0.001
+ Procedure × Surgeon	12780.1	98.9	0.027
+ Anesthesiologist	12770.6	108.5	0.001
+ Procedure × Anesthesiologist	12767.1	112.0	0.019
+ Surgeon × Anesthesiologist	12768.1	111.0	0.319
+ Circulator	12698.6	180.4	<0.001
+ Procedure × Circulator	12692.9	186.2	0.005
+ Surgeon × Circulator	12658.1	221.0	<0.001
+ Anesthesiologist × Circulator	12659.4	219.7	0.406

Table S15. Model selection for choosing random effects in the LMM where OR time is the response. The base model is given in Eq. (2) of the main paper and consists of a fixed intercept, all six fixed effects, and procedure as a random intercept. Additions appearing in this table are cumulative in the sense that each subsequent random effect was added to the model in the preceding row. AIC gain is the improvement in AIC from adding additional random effects onto the base model (calculated as AIC of the base model minus AIC of the larger model). Abbreviations: AIC, Akaike information criterion.

Model	AIC	AIC gain	p-value
Base model	21047.8	-	-
+ Surgeon	20062.6	985.2	<0.001
+ Procedure × Surgeon	19842.0	1205.7	<0.001
+ Anesthesiologist	19739.2	1308.6	<0.001
+ Procedure × Anesthesiologist	19720.5	1327.2	<0.001
+ Surgeon × Anesthesiologist	19694.6	1353.2	<0.001
+ Circulator	19641.8	1405.9	<0.001
+ Procedure × Circulator	19620.7	1427.1	<0.001
+ Surgeon × Circulator	19602.6	1445.1	<0.001
+ Anesthesiologist × Circulator	19599.6	1448.2	0.024

Table S16. Model selection for choosing fixed effects in the LMM where first case start time delay is the response. The base model consists of a fixed intercept, all six fixed effects, and the random effects found to be significant from Tab. S9. Each fixed effect was removed from the base model, and each reduced model was compared to the base model via a chi-squared test. If the base model was found significant compared to the reduced model, then the corresponding fixed effect was retained. Subtractions appearing in this table are not cumulative and denote that only the indicated fixed effect was removed from the base model and all other fixed effects were included. AIC loss is the increase in AIC from removing a fixed effect from the base model (calculated as AIC of the reduced model minus AIC of the base model). Abbreviations: AIC, Akaike information criterion; BM, base model.

Model	AIC	AIC loss	p-value
Base model	9830.7	-	-
BM − Number of procedures	9828.7	-2.0	0.919
BM − Number of panels	9828.7	-2.0	0.979
BM − Procedure level	9824.8	-5.9	0.989
BM − Cancer/noncancer	9831.7	1.0	0.081
BM − Position	9827.7	-3.0	0.598
BM − Patient class	9905.8	75.1	<0.001

Table S17. Model selection for choosing fixed effects in the LMM where setup duration is the response. The base model consists of a fixed intercept, all six fixed effects, and the random effects found to be significant from Tab. S10. Each fixed effect was removed from the base model, and each reduced model was compared to the base model via a chi-squared test. If the base model was found significant compared to the reduced model, then the corresponding fixed effect was retained. Subtractions appearing in this table are not cumulative and denote that only the indicated fixed effect was removed from the base model and all other fixed effects were included. AIC loss is the increase in AIC from removing a fixed effect from the base model (calculated as AIC of the reduced model minus AIC of the base model). Abbreviations: AIC, Akaike information criterion; BM, base model.

Model	AIC	AIC loss	p-value
Base model	11568.4	-	-
BM − Number of procedures	11596.8	28.4	<0.001
BM − Number of panels	11568.7	0.3	0.126
BM − Procedure level	11568.9	0.5	0.090
BM − Cancer/noncancer	11567.5	-0.9	0.300
BM − Position	11565.7	-2.7	0.517
BM − Patient class	11735.2	166.8	<0.001

Table S18. Model selection for choosing fixed effects in the LMM where anesthesia induction time is the response. The base model consists of a fixed intercept, all six fixed effects, and the random effects found to be significant from Tab. S11. Each fixed effect was removed from the base model, and each reduced model was compared to the base model via a chi-squared test. If the base model was found significant compared to the reduced model, then the corresponding fixed effect was retained. Subtractions appearing in this table are not cumulative and denote that only the indicated fixed effect was removed from the base model and all other fixed effects were included. AIC loss is the increase in AIC from removing a fixed effect from the base model (calculated as AIC of the reduced model minus AIC of the base model). Abbreviations: AIC, Akaike information criterion; BM, base model.

Model	AIC	AIC loss	p-value
Base model	13912.6	-	-
BM − Number of procedures	13967.0	54.3	<0.001
BM − Number of panels	13911.0	-1.6	0.549
BM − Procedure level	13912.2	-0.4	0.133
BM − Cancer/noncancer	13910.7	-2.0	0.912
BM − Position	13916.3	3.6	0.022
BM − Patient class	13983.9	71.3	<0.001

Table S19. Model selection for choosing fixed effects in the LMM where procedure start time delay is the response. The base model consists of a fixed intercept, all six fixed effects, and the random effects found to be significant from Tab. S12. Each fixed effect was removed from the base model, and each reduced model was compared to the base model via a chi-squared test. If the base model was found significant compared to the reduced model, then the corresponding fixed effect was retained. Subtractions appearing in this table are not cumulative and denote that only the indicated fixed effect was removed from the base model and all other fixed effects were included. AIC loss is the increase in AIC from removing a fixed effect from the base model (calculated as AIC of the reduced model minus AIC of the base model). Abbreviations: AIC, Akaike information criterion; BM, base model.

Model	AIC	AIC loss	p-value
Base model	25397.3	-	-
BM − Number of procedures	25442.1	44.9	<0.001
BM − Number of panels	25401.0	3.7	0.017
BM − Procedure level	25413.5	16.2	<0.001
BM − Cancer/noncancer	25395.6	-1.6	0.544
BM − Position	25394.9	-2.4	0.443
BM − Patient class	25509.4	112.1	<0.001

Table S20. Model selection for choosing fixed effects in the LMM where wheels out delay is the response. The base model consists of a fixed intercept, all six fixed effects, and the random effects found to be significant from Tab. S13. Each fixed effect was removed from the base model, and each reduced model was compared to the base model via a chi-squared test. If the base model was found significant compared to the reduced model, then the corresponding fixed effect was retained. Subtractions appearing in this table are not cumulative and denote that only the indicated fixed effect was removed from the base model and all other fixed effects were included. AIC loss is the increase in AIC from removing a fixed effect from the base model (calculated as AIC of the reduced model minus AIC of the base model). Abbreviations: AIC, Akaike information criterion; BM, base model.

Model	AIC	AIC loss	p-value
Base model	28228.0	-	-
BM − Number of procedures	28326.4	98.5	<0.001
BM − Number of panels	28226.0	-2.0	0.990
BM − Procedure level	28238.6	10.6	0.001
BM − Cancer/noncancer	28226.2	-1.8	0.651
BM − Position	28228.5	0.5	0.104
BM − Patient class	28414.3	186.3	<0.001

Table S21. Model selection for choosing fixed effects in the LMM where cleanup duration is the response. The base model consists of a fixed intercept, all six fixed effects, and the random effects found to be significant from Tab. S14. Each fixed effect was removed from the base model, and each reduced model was compared to the base model via a chi-squared test. If the base model was found significant compared to the reduced model, then the corresponding fixed effect was retained. Subtractions appearing in this table are not cumulative and denote that only the indicated fixed effect was removed from the base model and all other fixed effects were included. AIC loss is the increase in AIC from removing a fixed effect from the base model (calculated as AIC of the reduced model minus AIC of the base model). Abbreviations: AIC, Akaike information criterion; BM, base model.

Model	AIC	AIC loss	p-value
Base model	12656.1	-	-
BM − Number of procedures	12654.2	-1.9	0.771
BM − Number of panels	12654.8	-1.2	0.383
BM − Procedure level	12652.3	-3.8	0.525
BM − Cancer/noncancer	12660.2	4.1	0.013
BM − Position	12652.1	-4.0	0.990
BM − Patient class	12678.0	22.0	<0.001

Table S22. Model selection for choosing fixed effects in the LMM where OR time is the response. The base model consists of a fixed intercept, all six fixed effects, and the random effects found to be significant from Tab. S15. Each fixed effect was removed from the base model, and each reduced model was compared to the base model via a chi-squared test. If the base model was found significant compared to the reduced model, then the corresponding fixed effect was retained. Subtractions appearing in this table are not cumulative and denote that only the indicated fixed effect was removed from the base model and all other fixed effects were included. AIC loss is the increase in AIC from removing a fixed effect from the base model (calculated as AIC of the reduced model minus AIC of the base model). Abbreviations: AIC, Akaike information criterion; BM, base model.

Model	AIC	AIC loss	p-value
Base model	19599.6	-	-
BM − Number of procedures	21056.3	1456.7	<0.001
BM − Number of panels	19597.9	-1.6	0.542
BM − Procedure level	19696.7	97.1	<0.001
BM − Cancer/noncancer	19598.5	-1.0	0.323
BM − Position	19596.3	-3.3	0.688
BM − Patient class	19821.7	222.1	<0.001

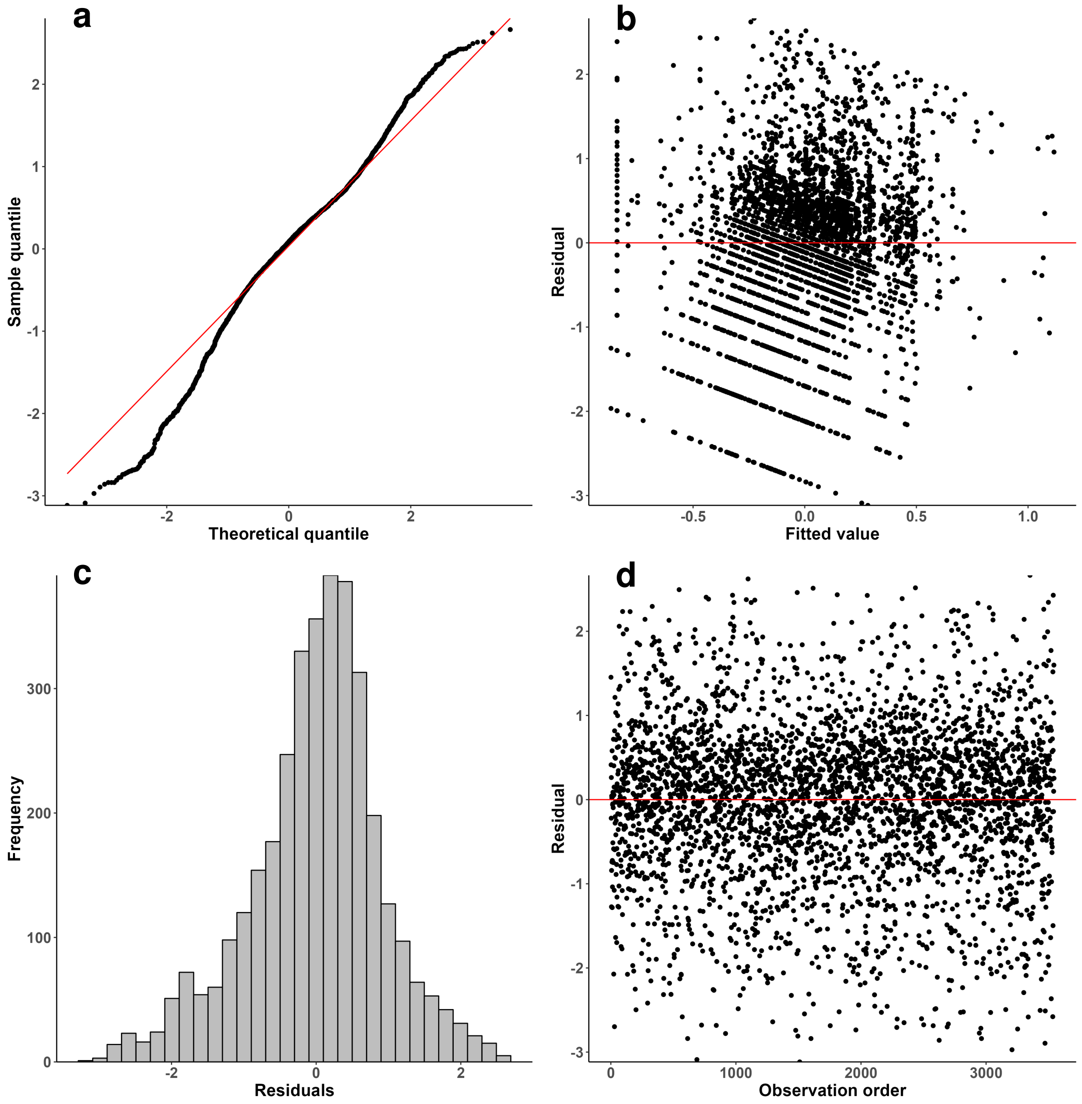

Figure S7: Diagnostic plots for the final LMM where first case start time delay is the response. (a) Normal probability plot of residuals; (b) residuals vs. fitted values; (c) histogram of residuals; (d) residuals vs. observation order.

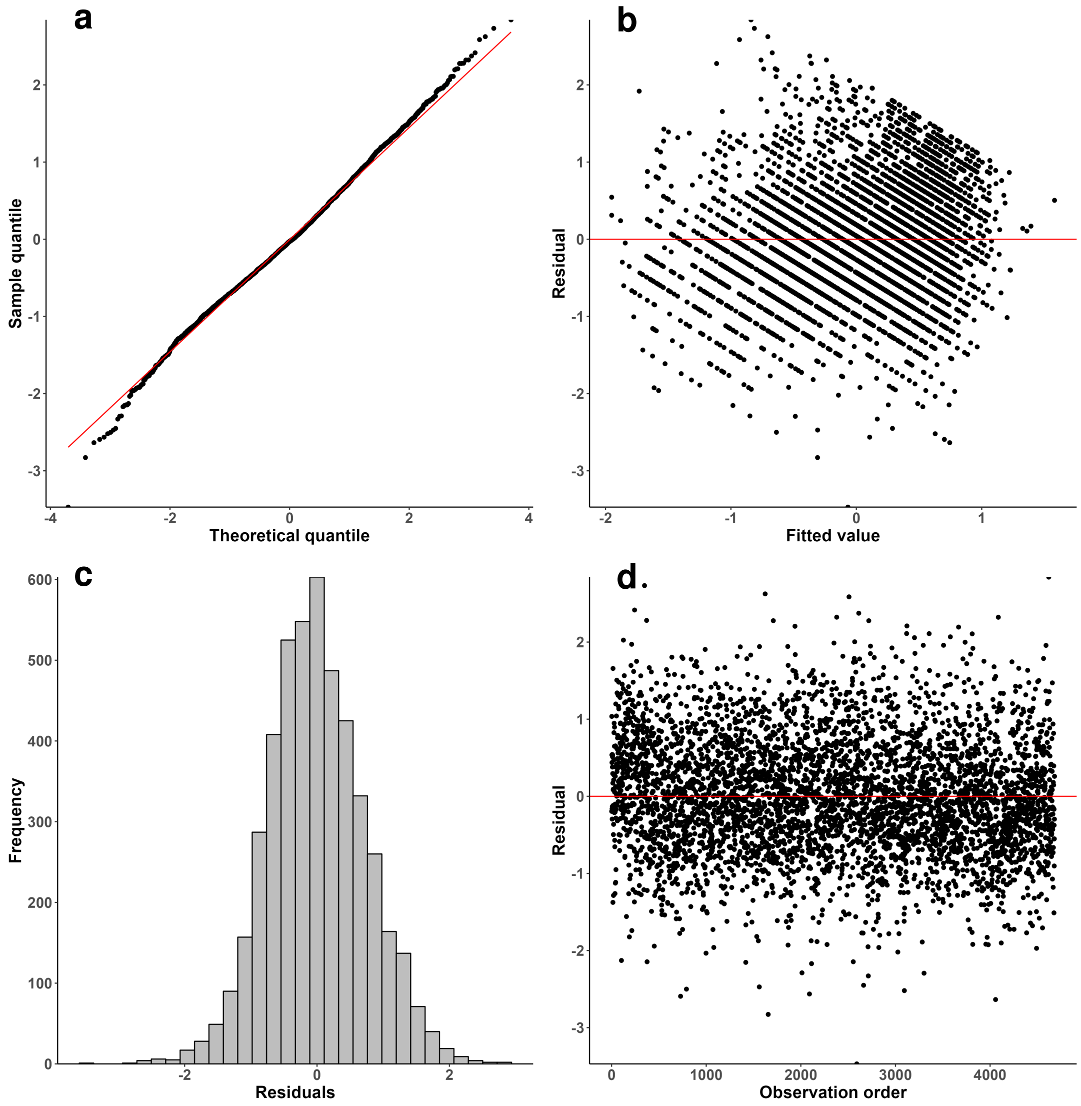

Figure S8: Diagnostic plots for the final LMM where setup duration is the response. (a) Normal probability plot of residuals; (b) residuals vs. fitted values; (c) histogram of residuals; (d) residuals vs. observation order.

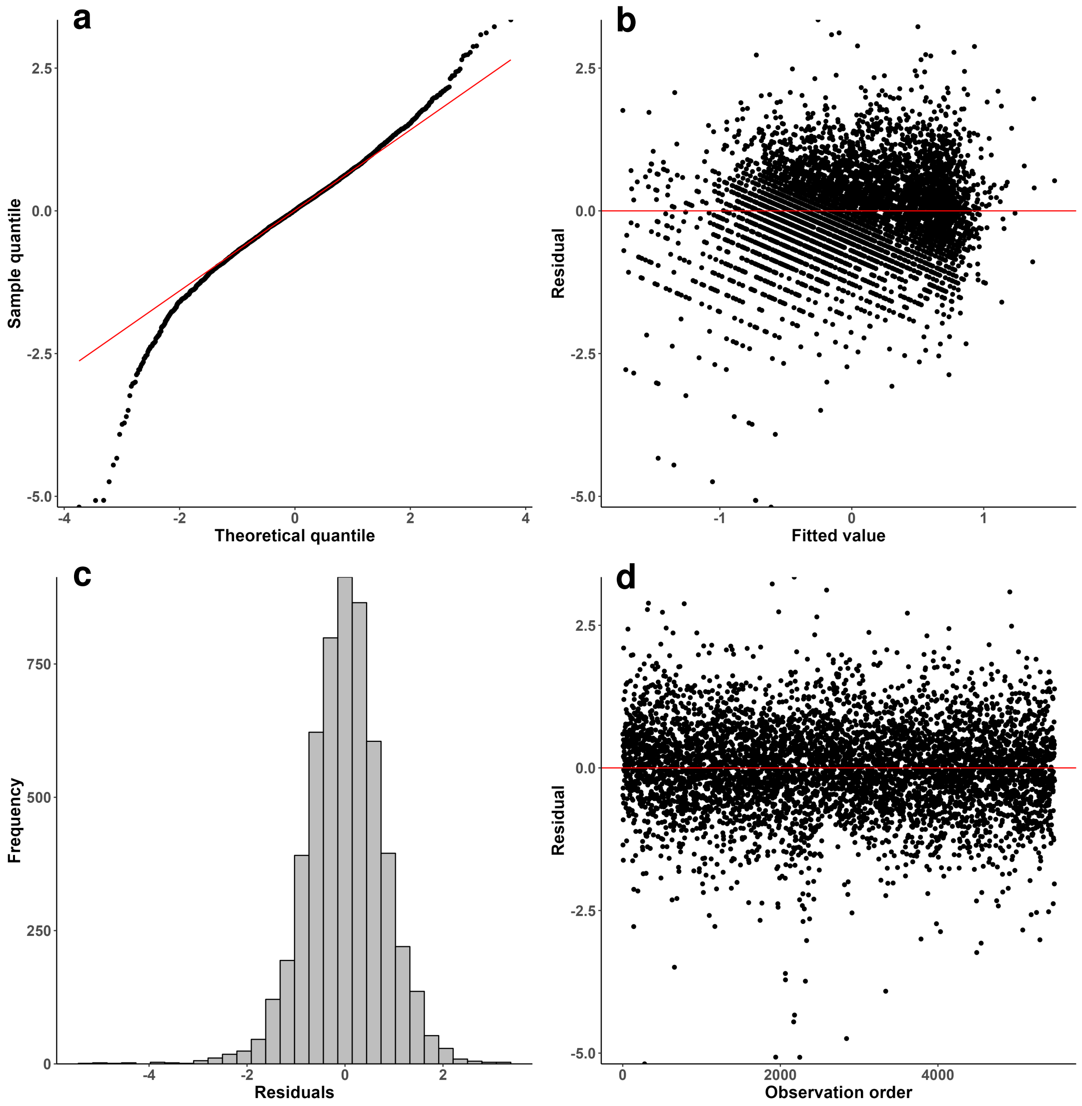

Figure S9: Diagnostic plots for the final LMM where anesthesia induction time is the response. (a) Normal probability plot of residuals; (b) residuals vs. fitted values; (c) histogram of residuals; (d) residuals vs. observation order.

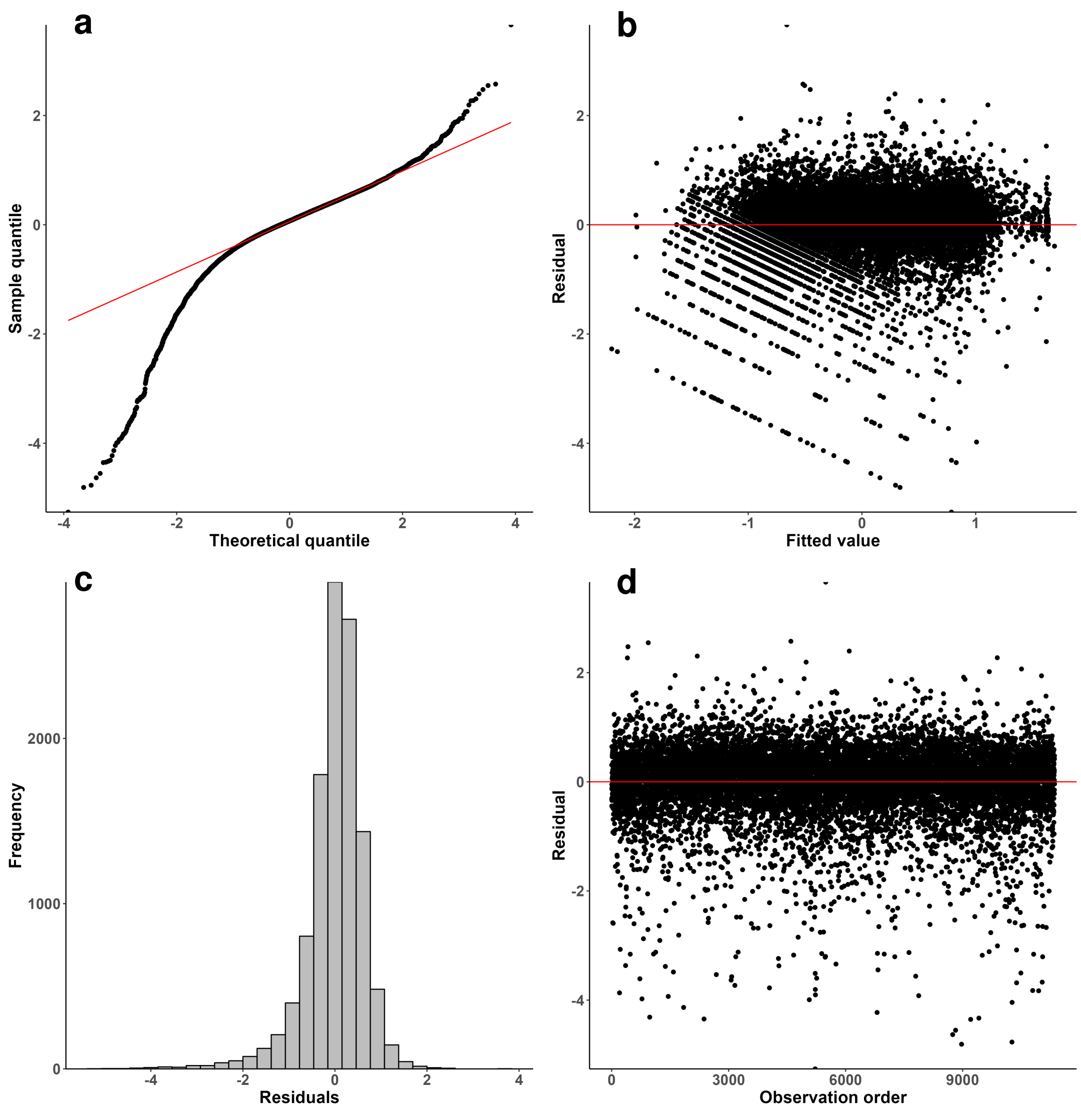

Figure S10: Diagnostic plots for the final LMM where procedure start time delay is the response. (a) Normal probability plot of residuals; (b) residuals vs. fitted values; (c) histogram of residuals; (d) residuals vs. observation order.

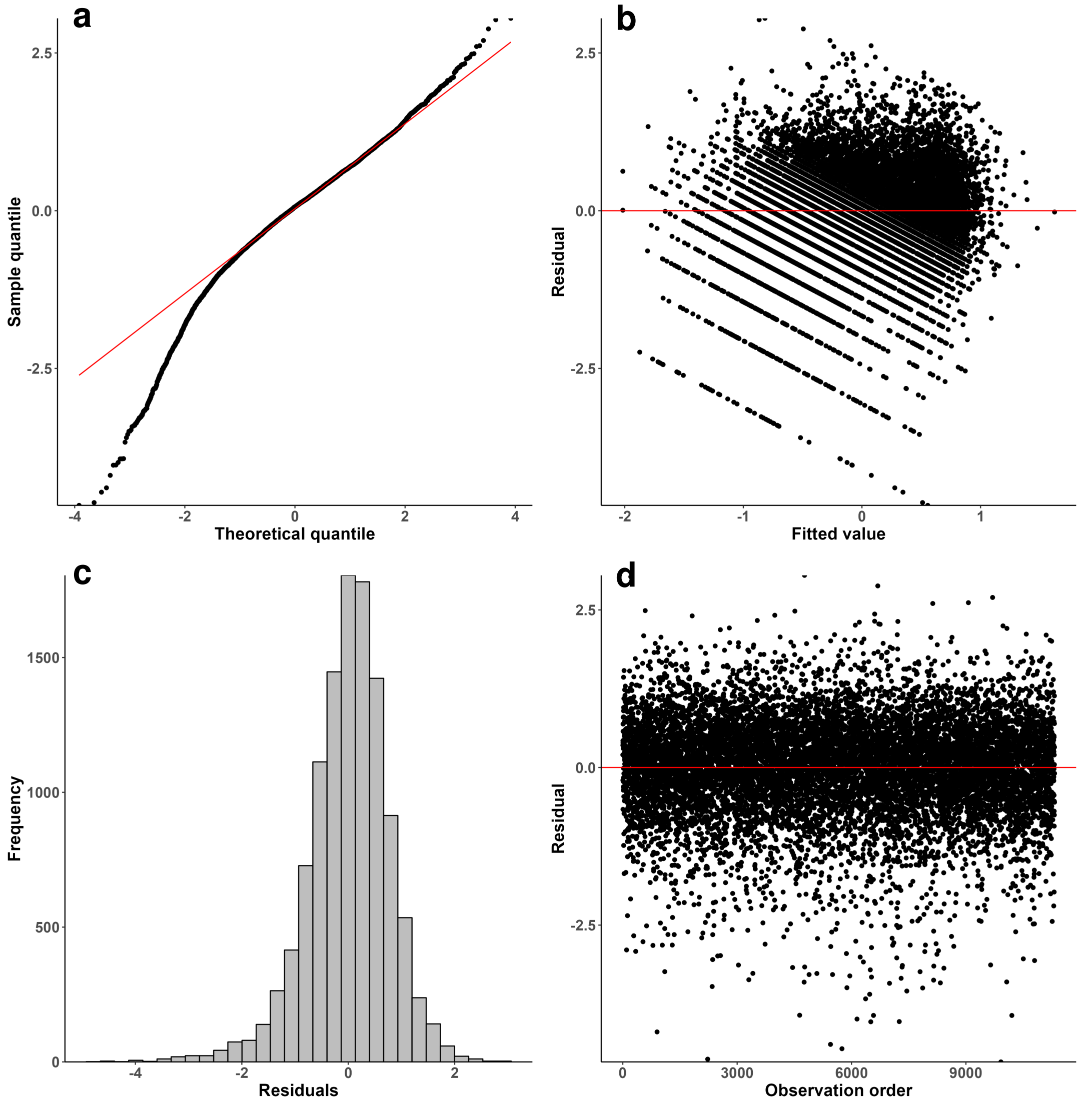

Figure S11: Diagnostic plots for the final LMM where wheels out delay is the response. (a) Normal probability plot of residuals; (b) residuals vs. fitted values; (c) histogram of residuals; (d) residuals vs. observation order.

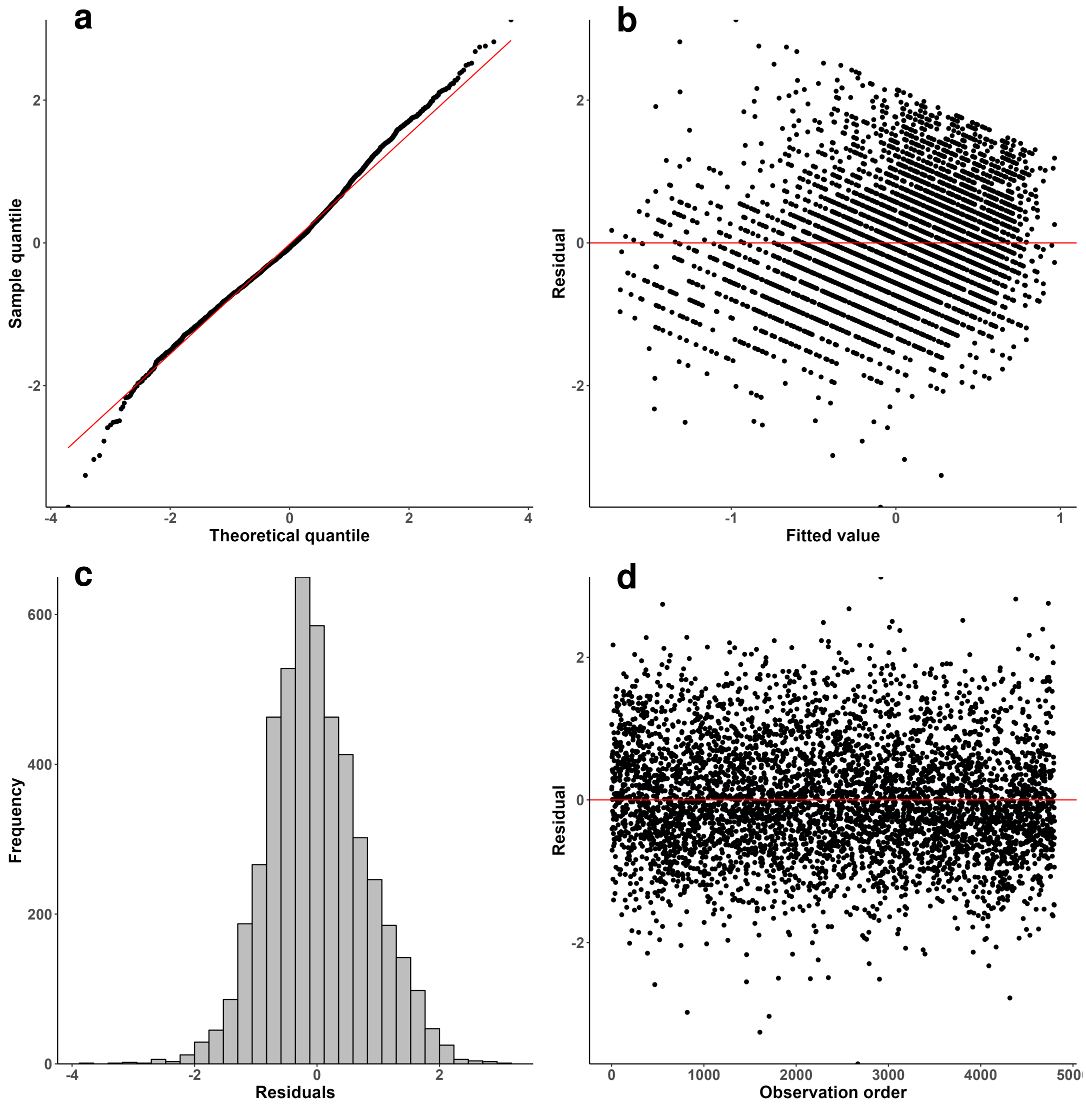

Figure S12: Diagnostic plots for the final LMM where cleanup duration is the response. (a) Normal probability plot of residuals; (b) residuals vs. fitted values; (c) histogram of residuals; (d) residuals vs. observation order.

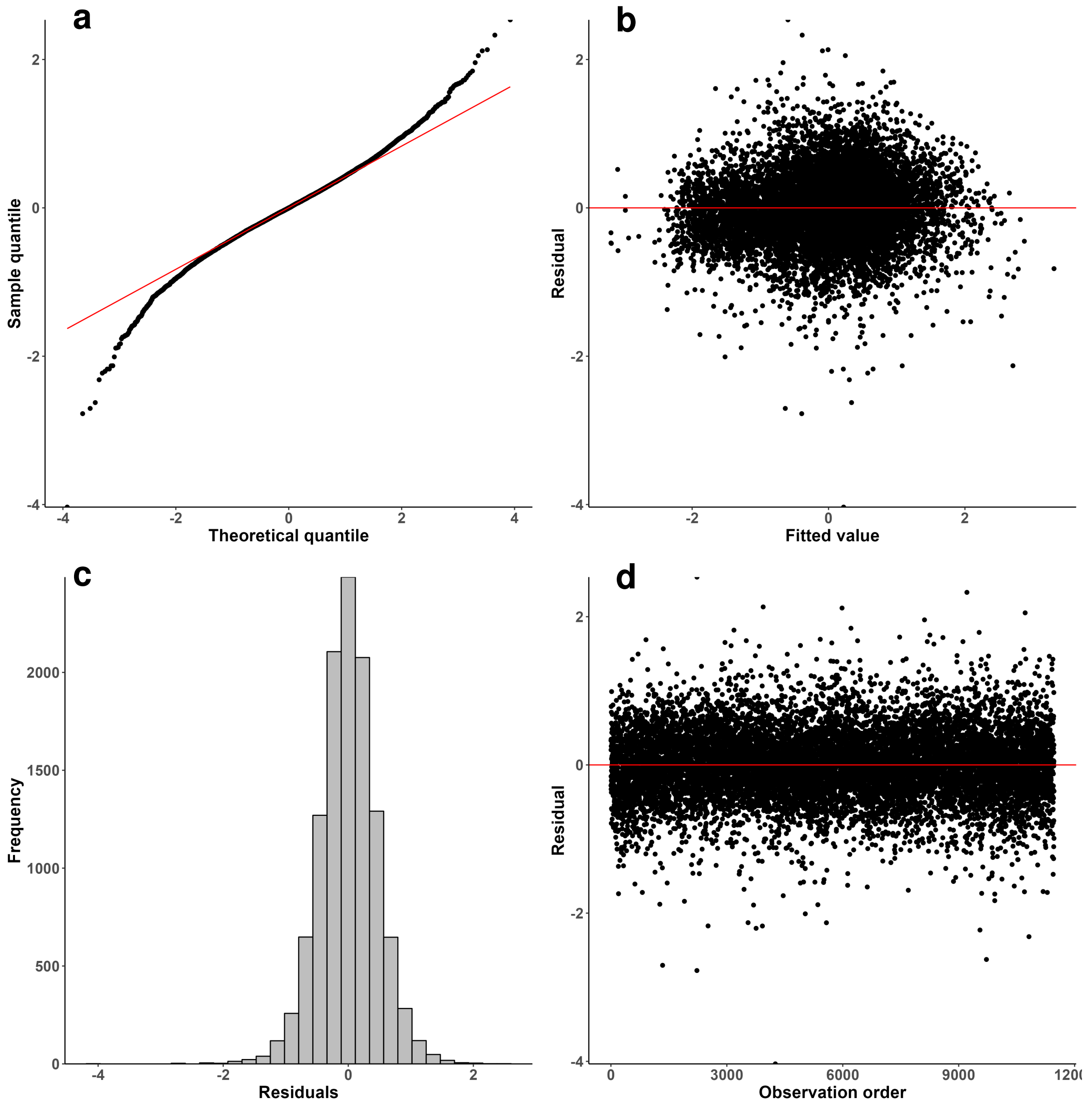

Figure S13: Diagnostic plots for the final LMM where OR time is the response. (a) Normal probability plot of residuals; (b) residuals vs. fitted values; (c) histogram of residuals; (d) residuals vs. observation order.
